# China’s freshwater lake storage hotspots revealed by bathymetry and typology mapping

**DOI:** 10.1093/nsr/nwag245

**Published:** 2026-04-25

**Authors:** Chunqiao Song, Kai Liu, Pengfei Zhan, Chenyu Fan, Weilong Yang, Liping Zhu, Bin Xue, Guoqing Zhang, Gang Zhao, Lian Feng, R Iestyn Woolway, Yunlin Zhang

**Affiliations:** State Key Laboratory of Lake and Watershed Science for Water Security, Nanjing Institute of Geography and Limnology, Chinese Academy of Sciences, Nanjing 211135, China; University of Chinese Academy of Sciences, Nanjing (UCASNJ), Nanjing 211135, China; University of Chinese Academy of Sciences, Beijing 100049, China; State Key Laboratory of Lake and Watershed Science for Water Security, Nanjing Institute of Geography and Limnology, Chinese Academy of Sciences, Nanjing 211135, China; University of Chinese Academy of Sciences, Nanjing (UCASNJ), Nanjing 211135, China; State Key Laboratory of Lake and Watershed Science for Water Security, Nanjing Institute of Geography and Limnology, Chinese Academy of Sciences, Nanjing 211135, China; State Key Laboratory of Lake and Watershed Science for Water Security, Nanjing Institute of Geography and Limnology, Chinese Academy of Sciences, Nanjing 211135, China; Natural Resources Survey, CGS, Beijing 100055, China; State Key Laboratory of Tibetan Plateau Earth System, Environment and Resources (TPESER), Institute of Tibetan Plateau Research, Chinese Academy of Sciences, Beijing 100101, China; State Key Laboratory of Lake and Watershed Science for Water Security, Nanjing Institute of Geography and Limnology, Chinese Academy of Sciences, Nanjing 211135, China; University of Chinese Academy of Sciences, Beijing 100049, China; State Key Laboratory of Tibetan Plateau Earth System, Environment and Resources (TPESER), Institute of Tibetan Plateau Research, Chinese Academy of Sciences, Beijing 100101, China; Key Laboratory of Biodiversity and Environment on the Qinghai–Tibetan Plateau, Ministry of Education, School of Ecology and Environment, Xizang University, Lhasa 850000, China; Key Laboratory of Water Cycle and Related Land Surface Processes, Institute of Geographic Sciences and Natural Resources Research, Chinese Academy of Sciences, Beijing 100101, China; State Key Laboratory of Information Engineering in Surveying, Mapping and Remote Sensing, Wuhan University, Wuhan 430079, China; School of Ocean Sciences, Bangor University, Anglesey LL57 2DG, UK; State Key Laboratory of Lake and Watershed Science for Water Security, Nanjing Institute of Geography and Limnology, Chinese Academy of Sciences, Nanjing 211135, China; University of Chinese Academy of Sciences, Nanjing (UCASNJ), Nanjing 211135, China; University of Chinese Academy of Sciences, Beijing 100049, China

**Keywords:** lake, China, freshwater resources, bathymetry, water depth, water storage

## Abstract

A nation’s freshwater security is fundamentally constrained by the total volume and spatial distribution of its water resources, yet this first-order information remains remarkably uncertain at large scales. For China, home to one-fifth of the world’s population but only 7% of its total renewable freshwater resources, this knowledge gap is particularly critical. While decades of satellite observations have tracked changes in lake surface area and water level, the total storage volume of the nation’s lakes have remained unknown, precluding a robust assessment of its water security. Here, we present the first national-scale bathymetric and typologic assessment of all natural lakes in China larger than 1 km². We reveal a total lake water volume of ∼1174 km³ (uncertainty range: 1081–1285 km³), comprising ∼335 km³ of freshwater and ∼839 km³ of saline water. While the western interior is numerically dominated by saline lakes, our volumetric assessment reveals a counterintuitive pattern in which a few large open lakes store ∼65% of the nation’s lacustrine freshwater due to their exceptional depth. The freshwater lake hotspots present a marked geographic–demographic contrast. This imbalance results in a more significant disparity in per-capita natural lake freshwater storage, with ∼20 680 m³ per person in the Tibetan Plateau lake zone versus only 65 m³ in the eastern plain lake zone, a nearly 320-fold difference. Notably, a vast network of reservoirs is concentrated in the populous eastern region, and this distribution of artificial storage helps mitigate the spatial unevenness of total surface freshwater resources. Our findings not only establish a definitive baseline for China’s lake water resources but also demonstrate how lakebed geomorphologic features influence national-scale lacustrine freshwater distribution, highlighting the critical need for targeted protection strategies and the efficient utilization of these lake freshwater reserves.

## Teaser

This study presents the first national-scale bathymetric and typologic assessment of China's natural lakes, revealing a total freshwater storage of approximately 335 km³. It is found that open deep-water lakes in the western interior store ∼65% of the nation's lacustrine freshwater, highlighting a profound geographic-demographic imbalance.

## INTRODUCTION

Safeguarding surface freshwater, one of Earth’s most essential yet limited natural resources, represents a defining challenge of the 21st century under escalating pressures from climate change and human activities [1–3]. Within the global water system, lakes serve as critical buffers by storing the most readily accessible liquid freshwater [4–6]. They thereby play a fundamental role in shaping regional hydrology, affecting biogeochemical cycling, and maintaining ecosystem stability [7–10]. Consequently, characterizing the key morphometric and typologic properties of lakes, principally involving their water depth, volume, and saline classification, is required to accurately assess water availability and improving Earth system models [7,11]. Lake depth, in particular, is a key controlling variable that regulates several physical attributes such as thermal stratification and ice phenology [12,13], as well as modulating key biogeochemical functions, including nutrient cycling, primary productivity, and methane emissions [14–18]. Distinguishing between freshwater and saline systems is equally important, as they

deliver different societal benefits and support unique ecosystems, with saline lakes offering distinct environmental and economic functions but not contributing directly to consumptive freshwater supplies [1,19].

Recent years have witnessed significant advances in lake bathymetric and typologic mapping, moving well beyond traditional, sparse field measurements [8,12,17,20,21]. Geo-statistical, fractal-based, and machine learning approaches now provide a robust theoretical framework for estimating lake depth and volume at broad scales by leveraging scaling relationships [7–9,17,22–26]. Additionally, the development of spaceborne photon-counting lidar has enabled direct bathymetric retrievals in clear water and inspired novel methods that infer depth from wave characteristics in turbid coastal waters where optical sensors fail [27–31]. Concurrently, a few research efforts have focused on classifying lakes as either freshwater or saline by integrating basin hydro-climate data and remote sensing spectral indicators of mineral deposits, achieving high accuracy of typologic classification [1]. Despite these scientific advances, a primary constraint remains, including the reliance on large-volume, high-quality training datasets of direct field measurements, which are often unavailable or skewed toward larger, accessible lakes [8,12,18]. This gap is particularly acute in vast territories such as China, where a historical lack of public surveying data has limited the accuracy of regional assessments on lake water storage within global models [7,8].

China exemplifies this water security predicament, facing a dual threat of quantity shortages and quality degradation [5,32,33]. While the eastern plains host high population densities, their shallow lakes are increasingly compromised by eutrophication and seasonal drying, rendering the identification of high-volume freshwater reserves critical for national strategic planning. This makes the nation’s thousands of lakes critically important assets [5,34–36]. Decades of remote sensing research have documented the dynamics of lake water area, level, and storage, revealing a strong geographical heterogeneity [37–39]. On the Tibetan Plateau (TP), a warming and wetting climate has driven a significant expansion of high-altitude lakes, whereas in the densely populated eastern plains and northern drylands, human activities have led to the shrinkage of many freshwater lakes [32,33,39–44]. These extensive one- or two-dimensional assessments, however, lack the crucial third dimension of water depth [31,42]. As a result, the nation’s actual volume of stored water cannot be assessed, leaving the true distribution and abundance of its resources unknown. This knowledge gap underscores the urgent need for a national-scale investigation to define this missing dimension.

To bridge this gap, our study provides nationwide bathymetric and typologic mapping of all 2713 natural lakes in China that are larger than 1 km². Our work is built upon a dataset compiled from two national-level initiatives, which provided field-surveyed depth and/or bathymetric measurements for 588 lakes, accounting for ∼78% and ∼87% of China’s total lake water area and storage, respectively (Text S1, Figs S1–S3, Tables S1–S10). For the remaining un-surveyed lakes, we developed and applied a suite of region-specific geo-statistical models tailored to the distinct geomorphological characteristics of China’s major lake zones (see Methods, Text S2). Furthermore, we classified all lakes as either freshwater or saline by delineating their drainage basin characteristics using the framework of integrating HydroBASINS, Lake-TopoCat, a lake-oriented drainage delineation method, and a vast amount of field-surveyed lake salinity data (see Methods, Text S3) [42,45–50]. By synthesizing these results, we deliver the first robust quantification of China’s lacustrine water resources. This work establishes a crucial baseline of the nation’s freshwater reserves, reveals previously overlooked hotspots of freshwater storage, and provides the essential data needed to formulate more resilient water allocation policies.

## RESULTS

### Lake depth over abundance reveals a sharp spatial contrast

Our circa-2020 national inventory reveals that China’s vast territory includes 2713 natural lakes greater than 1 km², which collectively cover a surface area of 79 892.4 km² (see Methods, Text S1). The distribution of these lakes is highly variable. Numerically, the inventory is dominated by smaller lakes, with 2029 lakes (75% of the total count) between 1 and 10 km² in size. Spatially, these lakes are primarily clustered in two major regions: the high-altitude TP and the low-lying eastern plains. Based on distinct geomorphological and climatic settings, China’s lakes were categorized into six major lacustrine regions [33,42]. The Tibetan Plateau lake-zone (TPL) contains 1187 lakes (44% of the national total count) and 56.6% of the total lake surface area (45 210 km²). In the east, a dense cluster of freshwater lakes are distributed, with the Eastern Plain lake-zone (EPL) and the Northeast Plain and Mountain lake-zone (NPML) hosting 481 (17.7%), and 415 (15.3%) lakes, respectively. In contrast, the arid regions, including the Mongolia Plateau lake-zone (MGPL, 352) and Xinjiang Plateau lake-zone (XJL, 222), are more sparsely populated with lakes, while the humid-subtropical Yunnan–Guizhou Plateau lake-zone (YGPL, 56) hosts the fewest lakes (Fig. S2).

**Figure 1.**
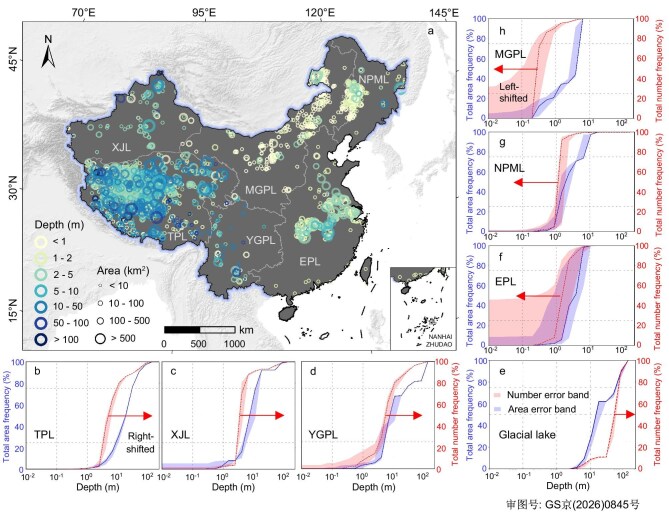
Map of lake water depth distribution (a) and zonal hypsometric models of statistical cumulative frequency of lake area and number varying with water depth (b–h). Six zonal divisions include NPML: Northeast Plain and Mountain lake-zone, MGPL: Mongolia Plateau lake-zone, XJL: Xinjiang Plateau lake-zone, TPL: Tibetan Plateau lake-zone, YGPL: Yunnan–Guizhou Plateau lake-zone, and EPL: Eastern Plain lake-zone. Field photos of lake depth sounding are shown at the top panel.

While the distribution of lakes shows a clear geographical imbalance, a more fundamental and contrasting spatial pattern emerges when we incorporate water depth. Our bathymetric mapping reveals a profound divide across the country. Deep lacustrine systems are concentrated in the western interior, whereas uniformly shallow lakes characterize the eastern and northern plains (Fig. 1a). The high-altitude west and southwest, specifically the regions surrounding the Himalayas, Kunlun, Tianshan, and Hengduan mountain ranges, form the deep structural basin depressions to house China’s deepest lakes. The TPL and YGPL lakes exhibit high mean depths of 21.3 m (20.5–22.7 m) and 24.9 m (23.5–26.3 m), respectively. This concentration of deep water is highlighted by the fact that the TPL alone contains 110 of the nation’s 128 deep lakes, which we defined here as those deeper than 40 m. Conversely, the lowlands of the east and north are characterized by exceptionally shallow basins. The EPL, NPML, and MGPL zones all feature average lake depths of <4 m (3.6 m (1.2–5.6 m), 3.5 m (3.0–4.1 m), and 3.2 m (2.5–3.9 m), respectively) (Fig. S4). Over 85% of the lakes in these three zones are less than 2 m deep. This pronounced east–west divide in lake depth, which is more striking than the distribution of lake number and area, reflects the fundamentally different geological and geomorphological processes that shape these lake beds. In the western interior, active rifting and high-relief mountain barriers create endorheic ‘sinks’, maintaining deep-water profiles. In contrast, the eastern plains occupy stable cratonic blocks within an exorheic system, where large riverine sediment supply rapidly infills basins, preventing the development of deep-water systems [51–53].

The regional patterns of lake depth are supported by clear signatures in their cumulative frequency distribution curves (Fig. 1b–h, Figs S5 and S6). A consistent feature across all zones is that the curve for lake number is positioned to the left of the area curve. It indicates that the most numerous lakes are systematically shallower than the larger lakes, which dominate the total surface area. These curves exhibit two primary styles reflecting underlying bathymetric characteristics. The first is a ‘gradually rising, right-shifted’ style, which is characteristic of deep-lake systems in the three western lake zones (TPL, XJL, and YGPL). For example, in the TPL, 50% of the total lake area is found in basins deeper than 19 m. The second style is a ‘steeply rising, left-shifted’ curve, found in the shallow-basin zones of the east and the north. This hyperbolic shape signifies a landscape dominated by uniformly shallow lakes. The MGPL represents such a typical case, where the shallowest 25% of total lake area exists in waters <1.2 m deep. Distinct from these two styles, the separate Glacial-lake group presents a unique signature where its cumulative number curve rises abruptly at great depths (40–80 m), indicating that a high proportion of numerous small lakes are exceptionally deep. These distinct statistical fingerprints suggest a profound hydrographic divide, revealed by bathymetric analysis across China.

### Freshwater lake storage hotspots observed in western interior basins

**Figure 2.**
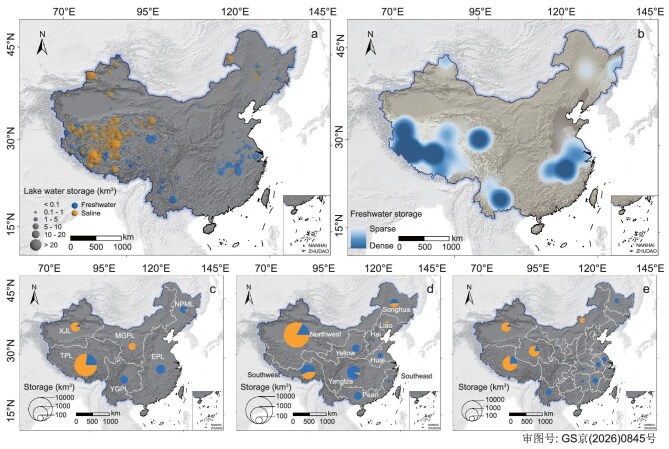
Map of spatial distribution of lake water storage across China and regional aggregation on freshwater and saline water storage. (a) Spatial distribution of freshwater and saline lake water storage. (b) Distribution of four freshwater lake hotspots (the western interior, including Taro Tso, Mapam Yumco, etc.; the Yellow River source region, including Eling Lake, Gyaring Lake, etc.; the southwest China, including Fuxian Lake, Dianchi Lake, etc.; and the eastern plains, including Poyang Lake, Dongting Lake, etc.) across China. (c–e) Regional aggregations of freshwater and saline lake water storage by lake zones, watersheds, and provinces, respectively.

China’s 2713 natural lakes (>1 km²) hold a vast water storage of ∼1174.3 km³ (uncertainty range: 1080.6–1284.8 km³). While the majority of this volume is chemically saline, an analysis of freshwater distribution reveals an unexpected hotspot in the deep lakes of the western interior (Fig. 2a). Freshwater storage (totaling 334.9 km³, uncertainty range: 277.5–388.7 km³) is clustered in two distinct regions: the populous eastern plains (for example, Poyang Lake and Dongting Lake) and, counterintuitively, in the high-altitude TP and its surroundings (Southwest and Northwest China). Contrary to the ‘saline west, fresh east’ view, the TPL contains the nation’s largest share of 215.2 km³ (206.2–230.4 km³), significantly surpassing the 65.0 km³ (20.8–99.2 km³) held by EPL. The western freshwater hotspots are supported by deep lakes, including the Taro Tso, Mapam Yumco, and Wuru Tso located in the southwestern TP, and the Eling and Gyaring Lakes in the northeastern TP (Fig. 2a, b).

Although freshwater constitutes the minority of total volume, with saline water dominating 71.5% (∼839 km³) of the national total (Fig. 2a), the spatial concentration of lake freshwater volume establishes the western interior as China’s primary storage hub. The TPL zone remains the country’s primary storage region overall, containing 963.6 km³ (82.1%) of the nation’s lake water storage. This volumetric dominance is driven by a unique combination of large, deep saline lakes, including the three largest lakes (Qinghai Lake, Siling Co, and Nam Co), which together store over 278 km³ (269–287 km³) of water, as well as the aforementioned deep freshwater lakes (Tables S11 and S12).

A hydrographic divide further characterizes these storage regimes. In the endorheic basins of the arid/semi-arid west, storage is overwhelmingly saline, with saline water comprising 78%, 87%, and 98% of the total lake volume in the TPL, XJL, and MGPL zones, respectively (Figs S7 and S8). The substantial volumetric dominance of western lakes is fundamentally governed by their specific geomorphological hosting mechanisms, wherein active tectonic subsidence along extensional fault systems and glacial overdeepening significantly outpace the rate of regional sediment infilling [8,52–54]. Conversely, the exorheic river systems of the monsoonal east and south, such as the Yangtze, Pearl, and Huai watersheds, are almost entirely fresh, with freshwater constituting 91%–100% of their lake water volumes. This partitioning reflects prevailing hydro-climatic regimes where the closed basins of the arid west accumulate salt, while the open and high-turnover hydrologic systems of the humid east maintain freshwater environments (Fig. 2c–e).

Our analysis refines conventional understanding by highlighting that the primary hotspot of lake freshwater volume is not the eastern plains, but the mountainous western interior. When combined with the deep lakes of the YGPL zone in Southwest China, the western region accounts for nearly three-quarters of China’s total lake freshwater storage. It is also underscored by the fact that 14 of the nation’s 20 most voluminous freshwater lakes, including the largest, Taro Tso, are located on the TP (Tables S11 and S12). This pattern is largely attributed to the exceptional depth of tectonically formed or glacially carved lakes due to the geological and geomorphological processes [12,13,51].

### Profound imbalance between lake freshwater geography and demography

**Figure 3.**
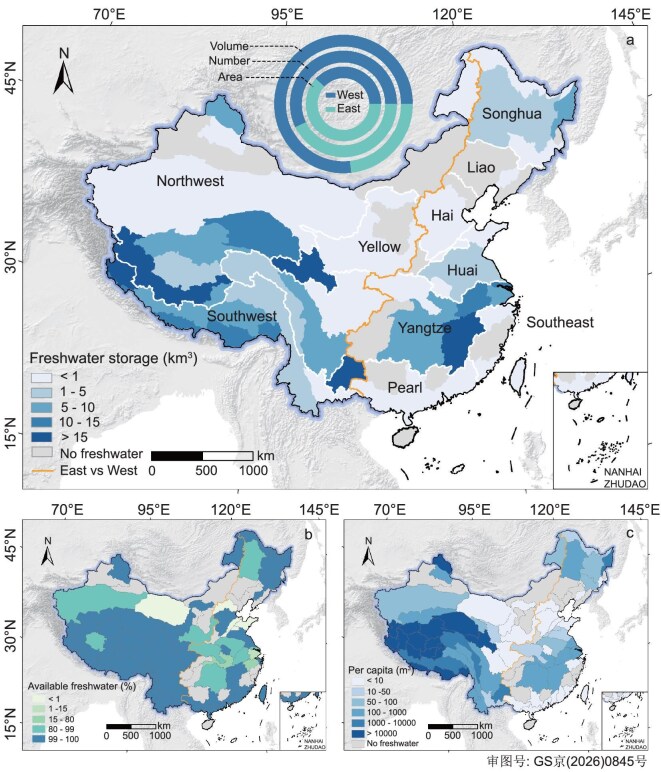
Contrasting pattern of lake freshwater stores across basins. (a) Map of freshwater lake storage by sub-basin. (b) Per capita available lake freshwater volume. (c) Proportion of ‘good-quality’ freshwater lake storage relative to the net storage after excluding moderate and hyper eutrophic lakes by watershed.

A profound spatial imbalance exists between where China’s lake freshwater is stored and where it is needed, a disparity quantified by a Gini index (a metric of inequality ranging from 0 [perfect equality] to 1 [maximal inequality]) of 0.79 for watershed-scale storage (Fig. 3a). The west, with just 38% of the nation’s freshwater lake area, holds a disproportionate 77% of the total volume (∼256 km³). Conversely, the populous east contains 62% of the lake area but only 23% of the freshwater volume (∼79 km³). This imbalance reflects the distinct topography of these lake basins: deep lakes of the western highlands inherently store far greater water volumes than the shallow fluvial lakes of the eastern plains. However, conventional per capita storage metrics often obscure the true extent of water stress by aggregating these disparate resources. The demographic contrast is evident, with 81% of the population in China residing in the east having access to only 23% of the freshwater lake storage [55], the average per capita availability is only 68 m³. In contrast, 913 m³ per person is available in the sparsely populated west, a nearly 13-fold difference (Fig. 3a, b). This contrasting pattern becomes even more pronounced when comparing the TPL zone (20 680 m³) to the EPL zone (65 m³), revealing a nearly 320-fold gap. However, the implications for water security are far more severe when the analysis shifts from ‘total storage’ to ‘accessible supply’ (see Methods, Fig. S9, Text S5). The immense TP freshwater reserves, while statistically significant, are largely decoupled from the nation’s primary agricultural and municipal demand centers due to extreme topographic barriers and high-altitude environments.

This geographic disparity is amplified by divergent hydrological trends that further widen the gap between storage and accessibility. Recent assessments reveal that the TP has witnessed a dramatic 20% expansion in lake area since the late 1990s, driven by increased precipitation and accelerated glacial melt [33,56,57]. However, these growing water reserves are largely trapped in remote and isolated basins, a significant proportion of this water is saline or brackish and economically unviable for direct transport to the eastern plains. In contrast, the densely populated eastern plains have experienced a net reduction in lake storage capacity due to intensive impoldering and consumption. Consequently, the ‘accessible supply’ defined as freshwater physically and economically available for distribution is significantly lower than the estimated ‘total storage’.

Furthermore, lake freshwater availability is increasingly constrained by widespread water quality degradation, which creates a compounding stress on the already scarce resources in eastern China. Eutrophication, driven by intense agriculture and urbanization, disproportionately affects the densely populated eastern lake basins. High-resolution monitoring of lakes in China from 1984 to 2023 indicates significant nationwide increases in trophic state index across eastern China [58], with severe eutrophication concentrated in the EPL zone (see Methods, Fig. 3c, Fig. S10). The crisis is particularly remarkable in the middle and lower Yangtze floodplain [58]. Analysis suggests that eutrophication in these key freshwater lakes result in a loss of accessible water volume estimated at ∼11 km³, a further reduction in per capita available water to 58 m³ across the eastern basin. This degradation effectively removes a large portion of the limited eastern water stocks from the potable supply chain. In the Taihu basin, for example, nearly 87% of lake freshwater resources are classified as poor quality, causing per capita available freshwater to descend from 115 to 14 m³ when the dimension of water quality is considered. In more critical watersheds such as the Hai River of the North China Plain, nearly 100% of the already scarce freshwater lake storage is of poor quality (Fig. 3c). Therefore, a robust assessment of China’s lake water security must integrate ‘quantity–quality-availability’, distinguishing between gross storage and the actual supply that meets safety standards for human and ecological health [4,5,41].

## DISCUSSION

### Uncertainty analyses of lake water storage and typology mapping

Our study establishes a ‘circa-2020’ baseline inventory, but it is essential to contextualize these findings within the bounds of statistical uncertainty. Specifically, the statistical lake water storage estimates are subject to upper and lower uncertainty bounds of 110.5 km^3^ (9.4%) and −93.7 km^3^ (8.0%), respectively. It is primarily contributed by the model prediction errors from a slightly higher proportion of unsurveyed TP basins and larger bathymetric scaling biases in the shallow eastern plain lakes (Fig. S39). While the Leave-One-Out Cross-Validation confirms robust predictive performance (*R*^2^ ranging 0.65–0.91) for the majority of zones, the reliance on geo-statistical inference for 78% of the lake count necessitates a cautious interpretation of local-scale figures, even though the national aggregate remains structurally dominated by the 87% of volume derived from direct field measurements.

The sources of this uncertainty are temporally and spatially heterogeneous. It is essential to acknowledge that the estimate of this study captures a specific phase in the multi-decadal evolution of China’s lakes. Historical remote sensing analysis reveals that over the past several decades, China’s total lake area increased by ∼9%, but with distinct regional heterogeneity [33]. Lakes in the TP and Xinjiang experienced significant expansions driven by accelerated glacial melt and increased precipitation, with a lake volume increase of 7–10 km^3^/yr, whereas the inner Mongolian Plateau and eastern plains saw reductions of 22% and 7%, respectively, largely due to warming-drying trends and human activities [33,43,56]. Consequently, our ‘circa-2020’ results likely capture a ‘highstand’ for western freshwater storage compared to the mid-20th century. While this implies that the current dominance of western lake freshwater is partly climate-driven and potentially transient on geological timescales, the substantial magnitude of the volumetric gap, where open deep-water lakes in the western interior hold ∼65% of national lacustrine freshwater, suggests that the contrasting storage pattern is a robust geomorphological feature that persists despite these decadal changes.

Seasonal dynamics, particularly in the monsoonal eastern plains, introduce another layer of uncertainty to static lake storage estimates. While our inventory utilizes a 25% water inundation frequency threshold to delineate relatively stable water bodies, estimating storage for floodplain lakes such as Poyang and Dongting Lakes remains challenging due to their drastic seasonally buffering hydrological regimes (Figs S39–S41, Text S4). Recent ICESat-2 altimetry studies indicate that while most lakes in China fluctuate by less than 1 m annually, these river-connected lakes can experience water level amplitudes exceeding 10 m [59,60]. Although some estimates suggest the annual throughput (flux) of Poyang Lake can exceed 100 km^3^, the actual seasonal storage variation is considerably smaller. National-scale assessments using laser altimetry estimate the total seasonal storage variation for all lakes across China as ∼75.5 km^3^ [59,60]. This seasonal amplitude represents ∼6.4% of the total static lake storage (∼1174 km^3^) identified in this study. Therefore, while seasonal fluctuations are critical for regional flood management, they do not fundamentally alter the national-scale conclusion that the deep western lakes serve as the primary static freshwater repository. Our sensitivity analysis (Text S4 and S5) demonstrates that the volumetric dominance of the western interior is determined by the geological and geomorphological processes rather than climatically transient. Even under a hypothetical scenario where eastern lakes reach maximum historical flood capacities and western lakes recede to the levels in the 1990s, the west would still hold >60% of the national freshwater storage due to the features of lake bathymetry and typology.

The binary classification of lakes into freshwater and saline categories relies on the nationwide field surveys and a methodological division of ‘open’ (outflow) vs. ‘closed’ (endorheic) basins. A rigorous validation using hydroclimate, spectral, and literature evidence on the TP demonstrated a classification accuracy of 94% in terms of lake area, confirming the method’s reliability for large water bodies [1]. However, it may inevitably introduce uncertainties due to the highly dynamic hydro-chemical processes currently being driven by rapid climate change and anthropogenic substance cycling (Text S3, Figs S32–S34). For instance, accelerated glacier mass loss and significantly increased precipitation across the TP and Northwest China have triggered a widespread and sustained ‘dilution effect’ [43,45]. This large influx of fresh meltwater is actively expanding lake volumes, profoundly altering the hydrological connectivity of closed basins, and potentially transitioning historically saline terminal lakes into brackish or even freshwater states over decadal timescales. Conversely, in the densely populated and agricultural basins of the Northeast Plain and eastern regions, intensive irrigation discharge and nutrient-laden runoff continuously exacerbate salt accumulation [47]. The high evaporation rates combined with these agricultural return flows gradually drive some morphologically open freshwater systems toward secondary salinization, a process already threatening the ecological integrity of major regional water bodies. These rapidly evolving biogeochemical trajectories suggest that our static, circa-2020 baseline estimates of freshwater storage may slightly underestimate the expanding freshwater resources in the western interior due to ongoing dilution, while concurrently overestimating the volume of chemically viable, pristine freshwater in the heavily cultivated and increasingly salinized eastern zones. This suggests that our classification of freshwater storage in the region might be conservative, as some lakes classified as ‘saline’ (based on topology) may currently be transitioning to brackish or fresh states. Thus, the classification uncertainty does not undermine the finding of a volumetric freshwater anomaly in the deep lakes of the west.

### Role of reservoirs in rebalancing the surface freshwater storage

The spatial configuration of China’s lake freshwater resources exhibits a geographic-demographic contrast. The TPL and YGPL zones are characterized by deep lakes, allowing for the storage of freshwater volumes that far exceed those of the eastern lowlands. In contrast, the EPL zone, despite its density of freshwater lakes, is featured by high infilling of riverine sediment and ‘saucer-shaped’ shallow basins. Consequently, 81% of China’s population resides in areas with direct access to only 23% of the nation’s natural lake freshwater storage. To meet the emerging demand of freshwater resources and to reduce the impact from floods and droughts [58,61–63], China has implemented a large engineering-based water security strategy. Over the past seven decades, the nation has constructed nearly 100 000 reservoirs [64,65]. These artificial reservoirs serve as the primary infrastructure for mitigating the severe spatial and temporal disparities in water availability [66].

**Figure 4.**
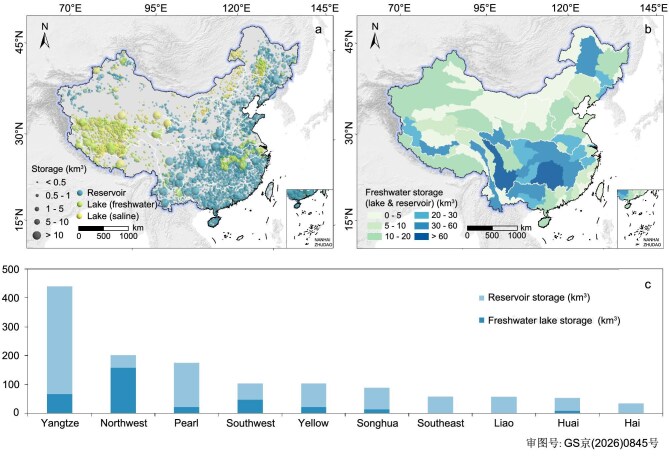
Spatial pattern and statistics on the alleviated inequality of freshwater stores by artificial reservoir impoundments. (a) The spatial distribution of reservoir and lake storage capacity. The spatial vector data of China’s reservoirs and associated storage capacity records are collected from the CRD database [64,65]. (b) The spatial distribution of freshwater storage (lake and reservoir) at the secondary basin level. (c) The ranked bar chart of freshwater storage summarized at the primary basin level.

The development of reservoirs has effectively buffered the spatial imbalance of natural lake freshwater. According to our bathymetric inventory and the CRD reservoir database, China’s artificial reservoirs provide a total storage capacity of ∼1000 km³, nearly triple the total natural lake freshwater storage (334.9 km^3^) [67] (Fig. 4, Fig. S11). These engineering-driven storage is spatially concentrated in the water-stressed eastern and southern monsoon regions (Fig. S12). The Yangtze and Pearl River basins alone hold over 54% of the national total capacity [68].

The network of artificial impoundments reduced the inequality of surface freshwater storage distribution. This rebalance is quantified by a significant reduction in the Gini index for total surface freshwater storage, which drops from 0.79 for natural lakes alone to 0.53 when reservoirs are included. In basins with minimal natural lake volume, reservoir impoundments have been transformative (Fig. 4a). For instance, the Southeast Rivers basin, with negligible natural lake storage, now commands ∼34 km³ of freshwater due to reservoirs (Fig. 4b, c). The combined lake-reservoir volume in the Yangtze (∼440 km³) now surpasses that of the lake-abundant Northwest basin (∼208 km³). This artifical storage has raised the total freshwater volume of the water-stressed east to levels that match the naturally water-rich west, with eight of China’s top 10 freshwater sub-basins now being reservoir-dominated.

### Implication for lake water resources management in China

The identification of freshwater lake hotspots through bathymetric and topologic mapping offers a critical reassessment of China’s surface freshwater storage. Our results indicate that the geographic disconnect between water resources and population centers serves as a functional safeguard for the nation’s freshwater reserves. For decades, lakes in eastern China have suffered from the combined pressures of water shortages and water quality degradation driven by agricultural intensification, urbanization, and industrial discharge. In contrast, the deep-water lakes identified in this study are located in high-altitude, remote regions of the west, largely isolated from major economic centers. This spatial separation has preserved the oligotrophic state of these large water bodies (for example, Taro Tso and Mapam Yumco), distinguishing them from the eutrophic waters common in the eastern plains. Consequently, the west functions as a high-quality strategic reserve. National policies should therefore prioritize the ecological preservation of these remote lakes rather than targeting them for immediate, extensive extraction.

Despite substantial engineering interventions, significant water deficits persist in the North China Plain (NCP) [69–71], Inner Mongolia [72], and part of northeast China [73,74]. Although recent decades have witnessed a warming and wetting trend in Northern China and the TP, increased precipitation alone cannot overcome the structural deficit of freshwater storage capacity. Unlike the western deep lakes, the shallow basins of the north lack the physical capacity to store significant additional volume. For instance, the Hai River basin holds only 5 km³ of lake freshwater storage, an amount insufficient to meet regional agricultural and industrial demands. This shortfall has necessitated heavy reliance on groundwater, leading to aquifer depletion, land subsidence, and ecosystem degradation. While the South-to-North Water Diversion Project (SNWDP) has helped mitigate depletion rates, long-term sustainability remains a challenge [75,76].

Ultimately, ensuring long-term water security requires integrating natural resources with engineered infrastructure. Our bathymetric data validates the volumetric potential of the western route of SNWDP but suggests that these western freshwater lakes may primarily serve as stabilizing node rather than primary supply sources in the national water network. We propose a dual-governance strategy: a conservation-oriented approach for the west, maintaining deep lakes as a protected strategic reserve, and an efficiency-oriented approach for the populous east. The latter should combine demand-side management with the conjunctive use of surface and groundwater, alongside the unconventional resources such as wastewater recycling and desalination. In an era of accelerating climate variability, this lake water storage-based assessment provides the necessary physical baseline for a resilient water security strategy, balancing western preservation with eastern regulatory approaches.

## METHODS

### National lake inventory and data sources

We constructed a comprehensive inventory of all 2713 natural lakes in China with a surface area >1 km^2^ (total area: 79 892 km^2^) for the baseline period circa-2020. This inventory integrates multi-source satellite altimetry, long-term field surveys, and geo-statistical modeling. Primary bathymetric data were derived from two national-level lake surveys conducted between 2010 and 2020 (NLS-2010 and NLS-2020). We obtained direct, field-surveyed depth measurements for 588 lakes using single-beam (200 kHz) and multi-beam echo sounders. While representing 21.7% of the numerical count, these surveyed lakes encompass 78.2% of the total national lake area and 87.3% of the estimated total storage volume, ensuring high confidence in the aggregate results.

### Bathymetric modeling and geo-statistics

For the 2101 lakes lacking direct field surveys (primarily remote, high-altitude, or small lakes), we developed region-specific geo-statistical models to estimate water volume. Lakes were categorized into six geomorphological zones (TPL, EPL, NPML, MGPL, XJL, and YGPL). We established empirical Area–Volume (A-V) scaling relationships (*V* = α *A^*β) for each zone using the surveyed lakes as calibration datasets. The adoption of a power-law A-V form is theoretically grounded in self-affine topographic scaling and has been empirically validated across diverse regions, providing a robust basis for regional parameterization [8,54,77]. The models achieved high predictive accuracy (Region-specific range: 0.65–0.91; see Text S2). Model uncertainty was quantified using Leave-One-Out Cross-Validation and propagated into the final estimates via Monte Carlo simulation.

### Typologic mapping and saline/freshwater lake classification

To quantify freshwater resources, lakes were classified as freshwater (TDS < 1 g/L) or saline (TDS ≥ 1 g/L). We employed a hierarchical classification framework integrating: (i) field-measured total dissolved solids (TDS) data for 588 lakes; (ii) Drainage topology analysis using the Lake-TopoCat database and HydroBASINS to identify endorheic (closed) vs. exorheic (open) basins; and (iii) Literature validation for un-surveyed large lakes. Lakes with surface outflows were classified as freshwater; endorheic lakes were classified as saline, with corrections applied for specific freshwater endorheic systems based on regional hydrogeological records (see Text S3).

### Volume estimation and uncertainty analysis

Lake water storage was calculated by integrating bathymetric grids (TIN interpolation) for surveyed lakes and applying A-V models for un-surveyed lakes. To address temporal variability, all lake surface areas were normalized to the circa-2020 reference period using Sentinel-2 water occurrence maps (threshold >25%). Total uncertainty (110.5 km^3^, −93.7 km^3^) was derived by aggregating measurement errors, model prediction intervals, and area extraction errors through a Monte Carlo framework (see Text S4).

## Supplementary Material

nwag245_Supplemental_File

## Data Availability

All analytical codes generated in this paper are available upon request (C. Song, email: cqsong@niglas.ac.cn). National inventory of lake water depth and storage estimates and tables of this article are publicly available at: https://doi.org/10.6084/m9.figshare.30082048.
