# Supplementary material for: China’s freshwater lake storage hotspots revealed by bathymetry and typology mapping
Source: Natl Sci Rev. 2026 Apr 25;13(9):nwag245. doi: 10.1093/nsr/nwag245 (PMC13188988; doi:10.1093/nsr/nwag245)
Supplement: nwag245_Supplemental_File [file nwag245_supplemental_file.pdf]

Supplementary Information for

China's freshwater lake storage hotspots revealed by bathymetry and typology mapping

Chunqiao Song[1,2,3*], Kai Liu[1,2*], Pengfei Zhan[1], Chenyu Fan[1], Weilong Yang[4], Liping Zhu[5], Bin Xue[1,3], Guoqing Zhang[5,6], Gang Zhao[7], Lian Feng[8], R. Iestyn Woolway[9], Yunlin Zhang[1,2,3*]

1. State Key Laboratory of Lake and Watershed Science for Water Security, Nanjing Institute of Geography and Limnology, Chinese Academy of Sciences, Nanjing 211135, China.
2. University of Chinese Academy of Sciences, Nanjing (UCASNJ), Nanjing 211135, China.
3. University of Chinese Academy of Sciences, Beijing 100049, China.
4. Natural Resources Survey, CGS, Beijing 100055, China
5. State Key Laboratory of Tibetan Plateau Earth System, Environment and Resources (TPESER), Institute of Tibetan Plateau Research, Chinese Academy of Sciences, Beijing 100101, China
6. Key Laboratory of Biodiversity and Environment on the Qinghai-Tibetan Plateau, Ministry of Education, School of Ecology and Environment, Xizang University, Lhasa 850000, China
7. Key Laboratory of Water Cycle and Related Land Surface Processes, Institute of Geographic Sciences and Natural Resources Research, CAS, Beijing 100101, China
8. State Key Laboratory of Information Engineering in Surveying, Mapping and Remote Sensing, Wuhan University, Wuhan 430079, China
9. School of Ocean Sciences, Bangor University, Anglesey LL57 2DG, UK.

* Corresponding to Y. Zhang (ylzhang@niglas.ac.cn), C. Song (cqsong@niglas.ac.cn), and K. Liu (kliu@niglas.ac.cn)

The file includes:

Supplementary Text S1 to S5

Supplementary Figure S1 to S41

Supplementary Table S1 to S12

Supplementary Text

Supplementary Text S1: Field survey and data harmonization

Lake area mapping

To delineate the lake water extent across China around 2020 (circa-2020), this study adopted a conventional and robust method for area extraction based on intra-annual water occurrence data [1-4]. This approach consists of three main steps: (1) constructing a spatial constrained dataset of lakes by extracting the maximum water inundation extent; (2) generating intra-annual water occurrence maps from multi-temporal satellite imagery; and (3) delineating lake water bodies using a compromise threshold applied to the water occurrence data.

Constructing a spatially-constrained lake inventory. In this study, lake area mapping was conducted with reference to a global dataset of maximum water extent developed by Liu et al. [3]. To ensure a clear and ecologically sound distinction between persistent lake ecosystems and strictly seasonal or ephemeral wetlands, the analytical framework defines the target lakes as topographically closed or semi-closed depressions that maintain a stable, permanent, or semi-permanent limnetic zone across inter-annual hydrological cycles. While temporary wetlands, such as seasonal floodplains, shallow coastal marshes, and transiently inundated agricultural zones, exhibit highly ephemeral inundation profiles driven strictly by short-term precipitation pulses, the target lake bodies sustain observable water extents even during prolonged dry seasons. This dataset characterizes the historical maximum extent of lake water bodies from 1984 to 2021 and was constructed by integrating the JRC Global Surface Water (GSW) product with the GLAD surface water dynamics data [5,6]. By clipping this dataset to the geographic boundary of China, we derived the maximum water extent of lakes within the country, which served as the basis for building a spatial constrain for subsequent analyses. In addition, to reduce potential errors and omissions inherent in a single data source, we incorporated lake inventory data from the 2023 national lake spatial catalog developed by the Ministry of Natural Resources of China for its nationwide lake water resources survey. Lakes missing from previous datasets were supplemented accordingly. As a result, a total of 2,713 lakes larger than 1 km^2were identified.

Water occurrence maps. Single-scene satellite imagery mapping is often affected by cloud and terrain shadow contamination and is inadequate for capturing the temporal variability of water bodies. To overcome these limitations, a water occurrence-based method was employed [4-6]. This approach enables more accurate and temporally representative delineation of water extent by integrating multiple observations over an extended period. In this study, all available high-quality Sentinel-2 imagery acquired during 2019 to 2021 was used, with scenes filtered using a <20% cloud cover threshold to ensure data quality. The use of a three-year image composite significantly increased the number of valid observations across China, yielding a median of 41 observations per

lake. Notably, nearly 85% of lakes had more than 10 valid observations, suggesting that the dataset provides sufficient temporal coverage to support reliable monitoring of lake dynamics.

All available Sentinel-2 imagery from the three-year period was composited to generate a representative dataset. This dataset characterizes the circa-2020 water inundation extents for each lake in the spatially constrained dataset described above, capturing intra-annual variations across China. To improve water body detection accuracy for each image scene, a well-established multi-index classification approach was employed [1,4]. This method integrates the Modified Normalized Difference Water Index (MNDWI), the Normalized Difference Vegetation Index (NDVI), and the Enhanced Vegetation Index (EVI) to reduce the influence of vegetation on water classification. A pixel was classified as water if it satisfied either the condition MNDWI > EVI or MNDWI > NDVI. To further eliminate residual interference from vegetation, an additional constraint of EVI < 0.1 was applied, thereby excluding mixed pixels containing both water and vegetation. The specific classification criteria are expressed as Equation (1-4) as follows:

$$MNDWI = \frac{Green - Swir}{Green + Swir} \tag{1}$$

$$NDVI = \frac{Nir - Red}{Nir + Red} \tag{2}$$

$$EVI = 2.5 \times \frac{Nir - Red}{Nir + 6 \times Red - 7.5 \times Blue + 1} \tag{3}$$

$$Water = \ (MNDWI > EVI\ or\ MNDWI > NDVI)\ and\ EVI < 0.1 \tag{4}$$

By accessing Sentinel-2 imagery covering the temporal span of the study area, water body detection was applied to all preprocessed scenes. Each image was subsequently converted into a binary classification map, where pixels were assigned values of 1 for water, 0 for non-water, and null for no observation. Based on the resulting binary maps, the total number of times each pixel was identified as water within the study area was calculated by summing the water-classified pixels (value = 1). In addition, the number of valid observations for both water and non-water pixels was computed. The water inundation frequency (WIF) map was then derived using the following Equation (5):

$$WIF = \frac{W}{F} \times 100\% \tag{5}$$

In this study, W denotes the number of water inundation for a given pixel during the specified period, and F represents the total number of valid observations for that pixel within the same timeframe.

Lake extent delineation based on water occurrence. To delineate the water extent of lakes in circa-2020, a frequency threshold of 25% was adopted [1,4]. This threshold corresponds to hydrological wet-dry transition conditions typically observed after the annually flooding season and serves as a proxy for assessing the water-holding capacity of lakes. Compared with lower

thresholds, such as 10% or 5%, the 25% threshold is less susceptible to the influence of short-term hydrometeorological anomalies and therefore provides a more robust representation of stable water bodies (**Fig. S13**). While it is acknowledged that higher frequency thresholds (e.g., >75% or >95%) are frequently utilized in remote sensing literature to isolate strictly permanent open water cores, the 25% threshold was deliberately selected as a vital eco-hydrological compromise. This specific threshold is uniquely capable of capturing the maximum stable geomorphological boundary of lakes characterized by severe intra-annual expansion and contraction, such as the highly regulated, seasonally buffering floodplain systems of the Yangtze River basin (e.g., Poyang and Dongting Lakes). Based on this procedure, the water extents of 2,713 lakes across China were successfully extracted for the circa-2020 period, yielding a cumulative surface area of 79,892.4 km^2.

Field data of lake water depth/bathymetry and storage

In this study, in-situ lake water depth and storage data were primarily obtained from three sources: two large-scale national lake surveys and a comprehensive collection of published literature reporting field measurements.

The first dataset originates from the **National Lake Survey conducted around 2010 (NLS-2010)** [7,8]. This survey primarily covered lake zones in eastern and northern China, with limited sampling over the Tibetan Plateau. During the campaign, more than 100 lakes were systematically surveyed using in-situ bathymetric measurements. After manual quality-control and data cleaning procedures, eventually 109 large and medium-sized lakes were kept and constituted a foundational dataset in the surveyed regions.

The second was a collection from the **National Lake Survey conducted around 2020 (NLS-2020)**. This project included a dedicated component on lake water resource assessment and conducted nationwide in-situ bathymetric surveys. Particular attention was given to collecting two categories of lakes: (1) those located in previously underrepresented regions, especially on the Tibetan Plateau, where historical data were scarce; and (2) representative small and medium-sized lakes with surface areas less than 50 km^2, which had previously been less surveyed. This data source provided a total of 244 lakes with measurements of bathymetric map and/or mean depth across China.

In addition to these two large-scale field surveying campaigns, we also compiled supplementary data on mean depth and storage from a wide range of sources with consistent quality controls, including **peer-reviewed literature, government bulletins, and credible media reports**. Although these values were originally derived from field measurements, the lack of standardized data-sharing mechanisms often limits access to the corresponding spatial bathymetric datasets. To ensure data quality and temporal consistency, we applied a rigorous data filtering and editing

procedure: records published before 2000 or associated with lake surface areas that deviated significantly from their 2020 extents were excluded. Through this data collection approach, we supplemented the data for a total 339 lakes in different lake zones, which are mostly medium- or small-sized.

By integrating data from the three sources described above, we compiled a comprehensive preliminary dataset of lake bathymetry and measured water depth and storage at the national scale. Among them, about 40 lakes were surveyed in both national lake investigations. In such cases, we prioritized the NLS-2020 dataset, owing to its use of more advanced survey technologies and its closer temporal alignment with the reference year of circa-2020 used in this study. To ensure consistency between survey campaigns, we conducted an inter-survey comparison for representative lakes after harmonizing the bathymetric data to a unified 2020 water extent (**Fig. S14**). The results show small elevation discrepancies between the two campaigns, with mean MAE and one-sigma errors of 1.04 m and 0.72 m, respectively, indicating good inter-survey consistency (**Fig. S15**). Similarly, for lakes with in-situ depth or bathymetric measurements, we did not collect the literature-reported data. Ultimately, a total of 588 lakes with in-situ water depth measurements were compiled across the country (**Table S1, Fig. S1**), including 69 from the NLS-2010, 209 from the NLS-2020, and 310 from published sources, which collectively account for **~78% of the total lake area** in China, contributing a **net water storage of approximately 1,025 km^3 (~87%)** by direct field measurements.

Workflow of field surveys for lake bathymetric measurements

Field survey planning

For obtaining representative water depth for lakes, the field surveying routes were generally designed based on each lake’s geomorphological features and hydro-meteorological conditions. Bathymetric transects were arranged perpendicular to the lake’s longest axis to ensure uniform spatial coverage. The spacing between adjacent transects does not exceed 500 meters, and each lake includes a minimum of three transects. To verify data quality and spatial consistency, cross-check lines perpendicular to the main transects were added, with their total length accounting for at least 5% of the primary survey lines (**Fig. S16**).

Lake depth measurements

Field data were collected using inflatable survey boats equipped with single-beam echo sounders. All operations strictly adhere to the industry standard Technical Regulations for Underwater Topographic Surveying in Inland Waters (CH/T 7003-2021), uniformly issued by the surveying project office. After completing the planned bathymetric transects, the accuracy and consistency

of the collected data are validated using the cross-check lines to ensure the overall reliability of the field survey.

Lake bathymetric mapping

Based on field surveyed data, three-dimensional underwater topography map was further constructed for each lake. The procedure involves three main steps:

(1) Bathymetric data preprocessing. Raw depth measurements were initially cleaned to remove outliers and ensure data quality. A Triangulated Irregular Network (TIN) was then constructed using the filtered bathymetric data points. The lake boundary, extracted from remote sensing imagery, is used as a spatial constraint during this process. The resulting TIN is subsequently converted into a raster format to create a continuous and gridded bathymetric surface (**Fig. S17**).

(2) Harmonization of underwater topographic extent. Due to the varying timeframes of field lake surveys, discrepancies may exist between the surveyed lake area and the referred circa-2020 lake extent adopted in this study. This issue was especially prominent for lakes that have experienced continuous expansion, where the surveyed area may be smaller than the waterbody area required for storage volume estimation.

To address this, for lakes where the surveyed extent was smaller than the circa-2020 lake extent, we first supplemented the unsurvey regions using elevation data from the SRTM DEM (acquired in February 2000). If the corresponding SRTM Water Body Dataset (SRTM SWBD) exceeds the area of the unsurvey region, we instead used the elevation data from the TanDEM-X DEM, acquired around the period 2011-2014, to ensure better spatial consistency and accuracy.

(3) Conversion between absolute elevation and water depth. An essential step in the integration process is reconciling the difference in vertical references: bathymetric DEMs derived from field surveys represent relative water depth, whereas DEMs such as SRTM and TanDEM-X provide absolute surface elevations. Harmonizing these datasets requires a transformation between depth and elevation. To estimate the lake surface elevation for indicating the circa-2020 status, elevation values along the remote-sensing-derived shoreline were extracted. After removing outliers beyond two standard deviations, the median of the remaining values was used as the reference water level basis for that year. A similar procedure was applied to determine the water level at the time of the field surveys [3,9]. Measured water depths were then adjusted by the difference between the two water levels to convert relative depths to absolute elevations. In unsurvey drawdown areas, water depth was estimated by subtracting the circa-2020 reference water level from the interpolated terrain elevation. This process yielded a harmonized water depth raster data for circa-2020, incorporating both field measurements and remote sensing–derived elevation data while accounting for temporal and spatial inconsistencies. The resulting dataset encompassed not only the largest and most prominent lakes in each lake zone (**Figs. S18-21**) but also a representative selection of medium and small-sized lakes (**Figs. S22-23**).

Supplementary Text S2: Geo-statistical modeling framework

Calculation of circa-2020 lake depth and storage based on field data

To estimate lake water depth and storage for the circa-2020 period, different approaches were applied depending on the availability of field-surveyed bathymetric data. For lakes with observed bathymetric measurements, the average water depth for each lake was derived by aggregating pixel-level depth values from this gridded bathymetric surface rather than directly averaging the original point measurements. Subsequently, water storage was calculated by estimating the water column volume for each pixel (pixel area × pixel depth) and summing all pixel volumes within the lake boundary. This spatially integrated approach ensures that both depth distribution and lake area are explicitly considered in the storage estimation. In total, 273 lakes were identified from the two national lake survey projects, encompassing a combined water surface area of 53,766.7 km^2, accounting for a percentage approximately 67% of national net area (**Table S1, Fig. S1**). The estimated total water storage for these lakes of this type reached 966.9 km^3(~82%).

For lakes with information of water depth and/or storage, yet lacking bathymetric map data, a correction-based estimation approach was adopted to derive the water depth and storage aligned with the circa-2020 reference year. Specifically, historical water depth and storage records were adjusted using multi-source satellite altimetry data (e.g., ICESat, ICESat-2) and contemporaneous optical imagery (e.g., Landsat-7/8, Sentinel-2) to construct area–volume–elevation (AVE) curves. These AVE curves enabled estimation of lake water storage at any time of observations, which was then corrected based on the lake surface area in circa-2020. The resulting estimates represent temporally harmonized water storage for the reference year. As a result, another 310 medium or small-sized lakes without direct bathymetric measurements, which contribute to ~11% of national net lake area, were assigned circa-2020 water depth/storage estimates, with a total calculated water storage of 58.6 km^3.

Estimation of water depth and storage for unmeasured lakes

Although many lakes lack field measurements and contribute only a small fraction of the national total water storage, it is necessary to develop statistical models for bathymetric prediction. These models leverage the spatial autocorrelation of lake area, depth, and storage with geographic and morphological features across different lake zones [10-12]. This approach involves constructing the statistical correlations between lake depth or water volume and variables such as lake morphology and surrounding topography within specific lake zones. By developing region-specific geostatistical models, we can estimate the water storage of lakes lacking direct measurements based on field-surveyed lake samples. The final nationwide lake water depth and storage can be estimated by integrating both lake area and model-based predicting models. Several

key issues were addressed during model development as follows.

Selection of modeling lake samples

Taking the EPL Region as an example, the area of lakes with field measurements ranges from 1 to 2400 km^2, whereas unmeasured lakes range from 1 to 80 km^2. Including all available field-surveyed lakes as modeling samples may skew it toward optimizing overall fit, compromising predictive accuracy for the specific range of lakes needing estimation. Therefore, the sample selection was determined by the upper area limit of unmeasured lakes within each region. For most lake zones, including EPL, only field-surveyed samples with surface areas between 1 and 100 km^2 were selected, as this range sufficiently represents the target group of unmeasured lakes. However, the Tibetan Plateau Lake (TPL) region presents a notable exception. In this region, a small proportion of unmeasured lakes (14) exceed 100 km^2 due to the difficult field-surveying conditions in the high-altitude plateau (**Fig. S24**). To ensure that the model adequately captures the variability of these larger lakes, lake with areas ranging from 1 to 400 km^2 were included in the sample set.

Construction of prediction models

By referring to prior studies [10-12], we adopted the form of a log–log regression model to establish the statistical relationship between lake storage (S) and area (A). Compared with multi-parameter polynomial models, the log–log linear model is simpler, involves fewer parameters, reduces the risk of overfitting, and generally achieves better overall accuracy (**Table S2, Figs. S25-30**). Theoretically, the prediction models were developed by lake-zone. However, two lake zones required special treatment due to their spatial heterogeneity. One is the Mongolian–Xinjiang Plateau Lake Zone, which spans a wide east–west gradient across northern China. Lakes in Inner Mongolia and neighboring provinces (e.g., Shaanxi, Gansu, and Ningxia) tend to be shallow and flat, while Xinjiang hosts many alpine lakes that are generally deeper. Therefore, this region was subdivided into XJL and MGPL subregions for separate modeling (**Figs. S27-28**). The other is the Tibetan Plateau Lake Zone, which contains numerous glacial lakes that are typically small in area but exceptionally deep. Applying a general model to these lakes would significantly underestimate their storage. In this study, we first identified 150 glacial lakes nationwide with areas greater than 1 km^2 based on existing glacial lake inventory [13]. We then collected all available in-situ records specific to glacial lakes and developed a dedicated estimation model for this lake type. We further identified certain lake types for which the lake-zone models were not fully representative of the statistical relationship between lake storage and area. For example, evaporative salty lakes in arid regions of western China often exhibit relatively large surface areas but shallow depths. For such cases, local models were constructed using in-situ samples from nearby lakes of similar type to better capture their characteristic S–A relationships. Using this strategy, the predicted storage of 81 lakes was revised. In addition, we

performed a separate validation analysis focusing on shallow lakes (mean depth < 2 m) to examine potential depth-dependent uncertainties (**Fig. S31**). Compared with glacial lakes, shallow lakes contribute only a small fraction to the total water storage and therefore have limited influence on absolute volume estimates. However, their relative errors exhibit greater variability, reflecting higher uncertainty in proportional terms within a narrow depth range.

Construction of the national lake water depth and storage dataset

By integrating both field measurements and model-based estimations, we developed a national-level dataset of lake water depth/storage for the year around 2020. In total, 588 lakes were supported by field-surveyed water depth observations (**Table S1**). Although these lakes represent only 21.7% of the total number of lakes included in this study, they account for as much as 87.3% of the total lake water storage in China. Notably, water storage estimates directly derived from measured bathymetric data collected during the two national lake surveys account for approximately 82% of the national total. Therefore, despite the numerical dominance of lakes without field data, nearly 90% of the national water storage is grounded in direct measurements, significantly enhancing the overall reliability of the storage estimates.

Further analysis based on lake surface area categories revealed that 90.1% of lakes larger than 100 km^2 were covered by field observations, with the field-measured area and water storage percentages up to 95.7% and 96.1%, respectively (**Tables S3-4**). The only notable exception was observed in the TPL region, where approximately 16% of large lakes (i.e., >100 km^2) lacked field-surveyed data due to their remote location in high-altitude, uninhabited areas with limited accessibility (**Fig. S24**). In contrast, other lake zones demonstrated comprehensive coverage, with nearly all large lakes possessing measured data. This is especially evident in the YGPL region, where in situ measurements were available for all lakes larger than 10 km^2, reflecting a high level of spatial data completeness for medium to large lakes (**Tables S5-10**).

Supplementary Text S3: Freshwater/saline lake classification

Typologic classification decision tree for lake typologic mapping

To further assess the spatial distribution of freshwater lake resources based on national-level lake water storage estimates, it is necessary to distinguish between saline and freshwater lakes across China. To achieve this, we developed and implemented a comprehensive two-tiered classification framework (**Fig. S33**).

We prioritized direct observational data by incorporating lake water salinity measurements from field investigations [14-16], including two national lake surveys. A total dissolved solids (TDS) threshold of 1 g/L was adopted: lakes with a TDS < 1 g/L were classified as freshwater, while those with a TDS ≥ 1 g/L were classified as saline. Prioritizing measured data directly addresses the limitations of relying solely on drainage topology, which can lead to misclassification in certain environments (**Fig. S33**). For example, in northeastern China (the NPML lake zone), intensive agricultural water consumption reduces effective recharge, while solute-rich agricultural runoff can cause salt accumulation, transitioning a morphologically open lake toward salinity or hypersalinity. Similarly, on the Tibetan Plateau, some through-flow lakes experience minimal discharge, leading to progressive salt accumulation over time.

For the remaining lakes lacking measured salinity data, we applied a second classification phase based on large-scale watershed runoff relationships and regional characteristics. First, all unclassified lakes were divided into endorheic and exorheic drainage basins [17-19] (**Fig. S34**). For lakes in endorheic regions, hydrological connectivity was further analyzed to differentiate between terminal and overflow lakes. We adopted a lake-oriented approach for lake drainage topology mapping[18,19]. This approach integrates DEM data and lake extent data to generate flow direction matrices, from which a preliminary drainage network is constructed and basic catchment units are delineated. To verify actual connectivity between potentially hydrologically-connected lake pairs, elevation profiles along the proposed flow paths are extracted. If no topographic peak exists between two lakes, they are considered connected by a natural drainage pathway; conversely, if a topographic high point is detected, the lakes are deemed disconnected and the link is removed. Following this correction, endorheic lakes with only inflow and no surface outflow were designated as terminal lakes and classified as saline. Lakes with both inflow and outflow connections were classified as overflow (through-flow) lakes and designated as freshwater. Based on the assumption that each terminal lake corresponds to a unique sub-basin, all first-order catchments draining into a terminal lake were aggregated to define its final sub-basin unit.

For lakes in exorheic regions, classification was determined by their specific geographic lake zones. Lakes situated in the EPL were directly classified as freshwater. For lakes located in the NPML and the YGPL, we relied on available scientific literature records to determine their saline

or freshwater status. Any exorheic lakes in these two specific regions that lacked literature records were classified as freshwater by default. As a result of this combined two-step approach, out of the 2,713 Chinese lakes included in this study, we categorized 1,182 as saline lakes and 1,531 as freshwater lakes.

Supplementary Text S4: Uncertainty quantification

Uncertainty analyses on lake water storage estimation

The uncertainty associated with the estimation of total water storage in Chinese lakes arises from three primary sources: field-surveying measurement errors, model-based prediction errors, and seasonal variations in lake storage.

(1) Field-surveying measurement uncertainty. For lakes with field-surveying measurements, lake bathymetry was typically reconstructed based on field-surveyed water depth data. Water storage is calculated by integrating the water column storage represented by each grid cell, using the Equation (6):

$$V = \int_0^A H(A)dA \tag{6}$$

In practical applications, this integral can be approximated as:

$$V = H * A \tag{7}$$

where V is the total lake water storage, H is the mean water depth, and A is the lake surface area. The uncertainty in the storage calculation (V_M) arises from two independent sources: water depth measurement error (σ_H) and lake area delineation error (σ_A). According to the law of error propagation:

$$\sigma_{V\_M}^2 = \sigma_{HA}^2 = \sigma_H^2 * \left(\frac{\partial V}{\partial H}\right)^2 + \sigma_A^2 * \left(\frac{\partial V}{\partial A}\right)^2$$

$$= \sigma_H^2 * A^2 + \sigma_A^2 * H^2 \tag{8}$$

$$\left(\frac{\sigma_{V\_M}}{V\_M}\right)^2 = \left(\frac{\sigma_{HA}}{HA}\right)^2 = \left(\frac{\sigma_H}{H}\right)^2 + \left(\frac{\sigma_A}{A}\right)^2 \tag{9}$$

The relative error is expressed as:

$$\frac{\sigma_{V\_M}}{V\_M} = \pm\sqrt{\left(\frac{\sigma_H}{H}\right)^2 + \left(\frac{\sigma_A}{A}\right)^2} \tag{10}$$

Consequently, the absolute uncertainty is calculated as:

$$\sigma_{V\_M} = \pm V\_M\sqrt{\left(\frac{\sigma_H}{H}\right)^2 + \left(\frac{\sigma_A}{A}\right)^2} \tag{11}$$

Mapping uncertainty from **Sentinel-2 imagery** arises primarily from its spatial resolution, particularly along lake shorelines where mixed boundary pixels occur. To quantify this uncertainty, we estimated the area error as one-half of the shoreline pixel area, assuming that the true boundary lies within edge pixels. This approach provides a concise and conservative estimate of lake area uncertainty in raster-based analyses (**Fig. S35**).

For bathymetric uncertainty, five lakes with independent check lines were selected within each study subregion (**Fig. S36-37**). By validating depth measurements for each lake using these check lines, we quantified the bathymetric measurement uncertainty at the individual lake level. Based

on these results, a representative bathymetric uncertainty was subsequently derived for each lake subregion (**Fig. S38**).

(2) Model-based estimation uncertainty. For lakes without field bathymetric surveys, water storage estimation relies on empirical models that relate lake storage to surface area and surrounding topographic factors. These models were developed using lakes with measured storages and applied to predict the storages of 2,101 unmeasured lakes. Model uncertainty is quantified via leave-one-out cross-validation. Given N observed lakes, the root-mean-square error (RMSE) of model predictions is expressed as:

$$\sigma_{V\_Test} = \pm\sqrt{\frac{1}{N}\sum_{n=1}^{N}(V\_Test_n - V\_True_n)^2} \quad (12)$$

where V_Test is the model-predicted storage and V_True is the observed water storage. The total uncertainty associated with the model-estimated storage (V_P) for all unmeasured lakes is calculated as:

$$\sigma_{V\_P} = \frac{\sigma_{V\_Test}}{V\_Test} V\_P \quad (13)$$

(3) Seasonal variability in lake storage. To quantify the uncertainty induced by seasonal dynamics of lake water storage, this study employed the estimates based on ICESat-2 altimetry observations from 2019 to 2021 and machine learning method[20], focusing on lakes larger than 1 km^2. Seasonal water level fluctuations were analyzed spatially across different basins and lake zones. Subsequently, seven hydrometeorological indicators (total precipitation, 2 m air temperature, runoff, surface runoff, total evaporation, potential evaporation, and open water evaporation) and reanalysis climate datasets (e.g., GRACE-FO) were integrated using machine learning algorithms to reconstruct and predict seasonal water level variations. Finally, seasonal water storage variability for all lakes was estimated by coupling water level fluctuations with corresponding lake surface areas. We estimated the aggregate seasonal water storage amplitude for lakes across China to be 75.5 km^3. The Yangtze River basin exhibits the most intense hydrological seasonality, driven largely by floodplain dynamics. Specifically, Poyang Lake and Dongting Lake serve as the primary regulators, registering seasonal storage amplitudes of 21.9 km^3 and 9.0 km^3, respectively (**Figs. S39c, S40-41**). Collectively, these two lakes account for approximately 41% of the national total seasonal variation, highlighting their critical role in buffering seasonal hydrological fluxes.

(4) Total uncertainty in total lake storage. For each lake zone, the total water storage uncertainty integrates the uncertainties from measured lakes (V_M), model-predicted lakes (V_P), and seasonal variations (σ_{V_S}) as follows:

$$\sigma_V^2 = \sigma_{V\_M}^2 \left(\frac{\partial V}{\partial V\_M}\right)^2 + \sigma_{V\_P}^2 \left(\frac{\partial V}{\partial V\_P}\right)^2 + \sigma_{V\_S}^2 \left(\frac{\partial V}{\partial V\_S}\right)^2 \quad (14)$$

$$\sigma_V = \pm\sqrt{\sigma_{V\_M}^2 + \sigma_{V\_P}^2 + \sigma_{V\_S}^2} \quad (15)$$

Beyond seasonal fluctuations, we assessed multi-decadal storage trends by synthesizing estimates from recent studies (Xu et al., 2022; Zhang et al., 2021). Over the period from the early 2000s to ~2020, China's lakes experienced a substantial net storage increase of 221.7 km^3. This expansion was spatially heterogeneous, with the Tibetan Plateau contributing ~186.5 km^3 (84% of the total), driven primarily by the expansion of Qinghai Lake (+16.9 km^3) and Siling Co (+16.0 km^3).Crucially, it is worth noting that the uncertainty profiles presented in **Fig. S39a–c** represent a composite metric accounting for three distinct sources of variance: field-measured uncertainties, modeled uncertainties, and the magnitude of seasonal storage variability. However, these intervals do not encompass long-term interannual trends. In other words, interannual variability was not explicitly incorporated into the 2020 storage uncertainty range. Therefore, when applying this dataset to estimate lake storage for years beyond the circa-2020 baseline, the corresponding interannual storage anomalies relative to 2020 (**Fig. S39d**) should be explicitly accounted for. These baseline shifts must be superimposed onto the seasonal cycle to ensure temporally consistent and accurate volumetric estimation.

Supplementary Text S5: Other auxiliary materials

Gridded population data

We analyzed the spatial distribution of population across China by utilizing the 2020 gridded population data from the GlobPOP dataset[21]. This dataset provides global population counts and densities at a 30-arc-second (~1 km) resolution, generated by fusing five widely used 1-km population products (GPWv4, GHS-POP, GRUMP, LandScan, and WorldPop) through a two-stage procedure. Specifically, (1) country-specific error diagnostics identify the optimal input data source in different regions; and (2) a quantile regression model blends these “best” pixels while constraining national totals to the UN World Population Prospects 2022 ds. Cross-validation demonstrates that the fused product explains 99.9% of national census variation (RMSE $\approx 1.7\times10^5$ people) and 97.8% of subnational variation (RMSE $\approx 2.6\times10^5$ people), outperforming any individual source raster. Population statistics and densities at China’s sub-basin scale (**Fig. S26**) indicate that, as of 2020, the eastern region of China was home to approximately 1,161 million people, accounting for about 80% of the national population, whereas the western region had only 280 million residents. All sub-basins in the east had populations exceeding one million, whereas more than half of the western watersheds had populations below one million. In terms of population density, a distinct east-to-west decreasing gradient is observed, with most western regions having densities below 100 people/km^2.

Lake’s Trophic State Index (TSI) data

The water quality data were obtained from the China Lake Trophic State Index (CNLTSI) dataset developed by Hu et al.[22]. The Trophic State Index (TSI) serves as a key indicator for quantifying and assessing lake eutrophication. This dataset derives the algal biomass index (ABI) from Landsat imagery and establishes ABI-TSI inversion models tailored to different types of water bodies. It provides annual average TSI for 2,693 lakes over 1 km^2 in China from 1984 to 2023. The estimated annual average TSI shows a coefficient of determination (R^2) of 0.98 when compared with in-situ measurements and collected validation data. In the TSI range of 0–100, five trophic state levels are defined: oligotrophic (TSI = 0–30), mesotrophic (TSI = 30–50), light eutrophic (TSI = 50–60), moderate eutrophic (TSI = 60–70), and hyper eutrophic (TSI = 70–100).

In this study, the mean TSI value from 2019 to 2021 was used to represent the trophic state of lakes in circa-2020. Among the 1,669 freshwater lakes analyzed, This TSI data were successfully matched for 1,581 lakes in this study (**Fig. S27**). For the remaining 88 unmatched lakes, the TSI value of the nearest lake to their centroid was used as a rough substitute. Approximately 80% of the lakes exhibited good trophic conditions, falling into the oligotrophic (46.5%) and mesotrophic (30.6%). These lakes are primarily distributed in the Tibetan Plateau lake-zone (TPL) and the

Yunnan-Guizhou Plateau lake-zone (YGPL). In contrast, moderate eutrophic and hyper eutrophic lakes account for 9.6% of the total and are mainly concentrated in the Yichang–Hukou Basin, the Taihu Basin, the Dongting Lake Basin, and the Nenjiang Basin. Among the moderate eutrophic lakes, two have water volumes exceeding 10×10^8 m^3, while all seven hyper eutrophic lakes have water volumes less than 1×10^8 m^3.

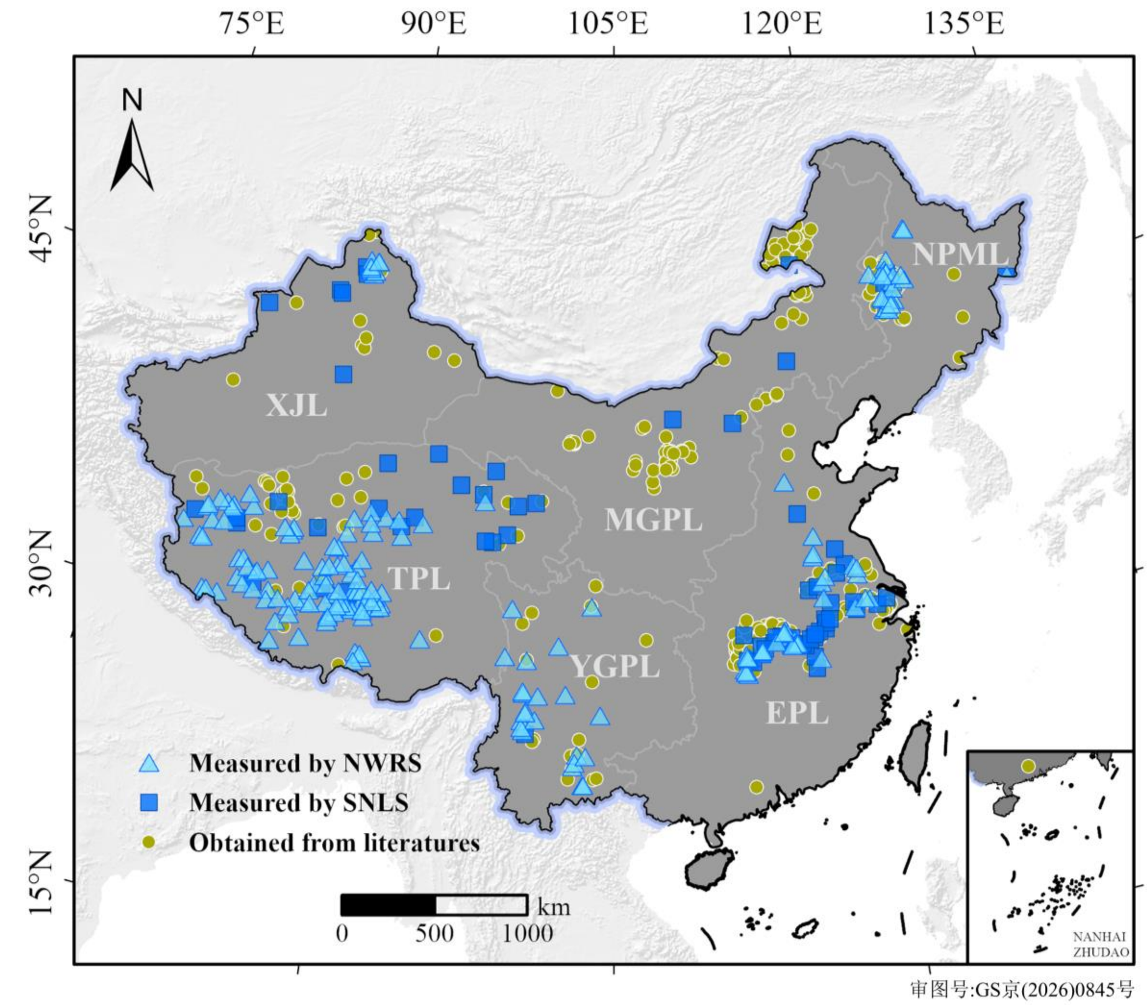

Fig. S1. Spatial distribution of lakes with available field-surveyed depth or bathymetric data. The data sources include National Lake Survey conducted around 2010 (NLS-2010) and around 2020 (NLS-2020), supplemented by additional samples collected from published literature (NPML: Northeast Plain and Mountain lake zone, MGPL: Mongolia–Xinjiang Plateau lake zone (excluding the Xinjiang area), XJL: Mongolia–Xinjiang Plateau lake zone (Xinjiang area), TPL: Tibetan Plateau lake zone, YGPL: Yunnan–Guizhou Plateau lake zone, EPL: Eastern Plain lake zone).

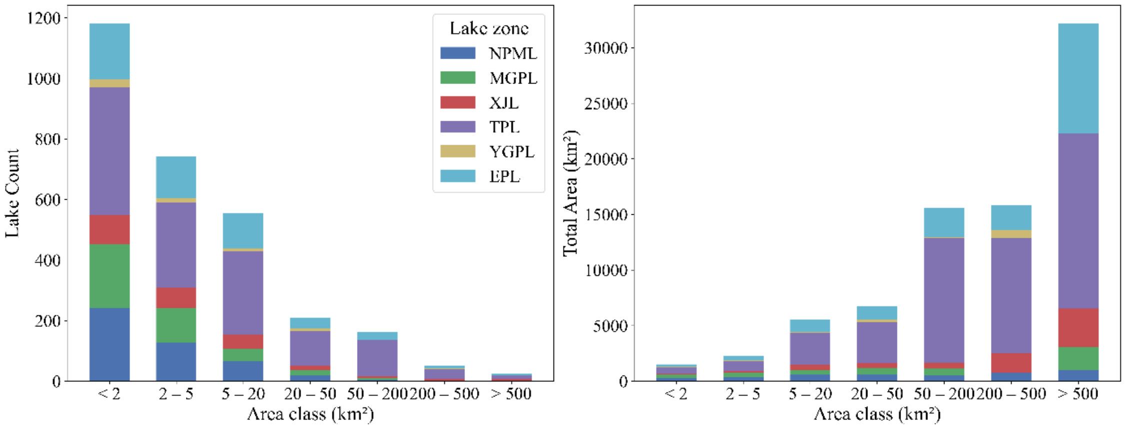

Fig. S2. Stacked distributions of lake count (a) and total lake area (b) across lake surface area classes, partitioned by lake zones (NPML, MGPL, XJL, TPL, YGPL, EPL).

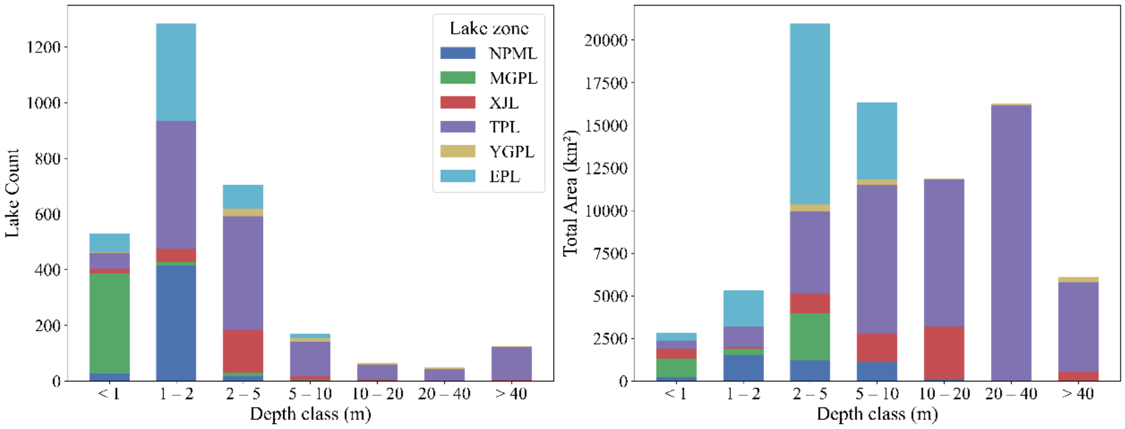

Fig. S3. Stacked distributions of lake count (a) and total lake area (b) across different depth classes, partitioned by lake zones (NPML, MGPL, XJL, TPL, YGPL, EPL).

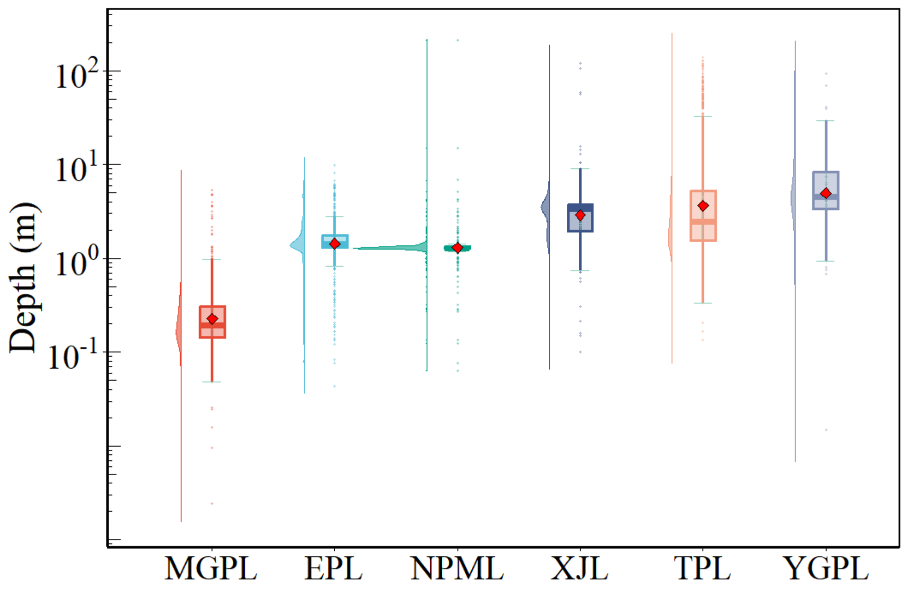

Fig. S4. Statistical distribution of lake depths in different lake zones. In the boxplots, red diamonds indicate the mean lake depth within each zone, while the horizontal lines within the boxes represent the median lake depth.

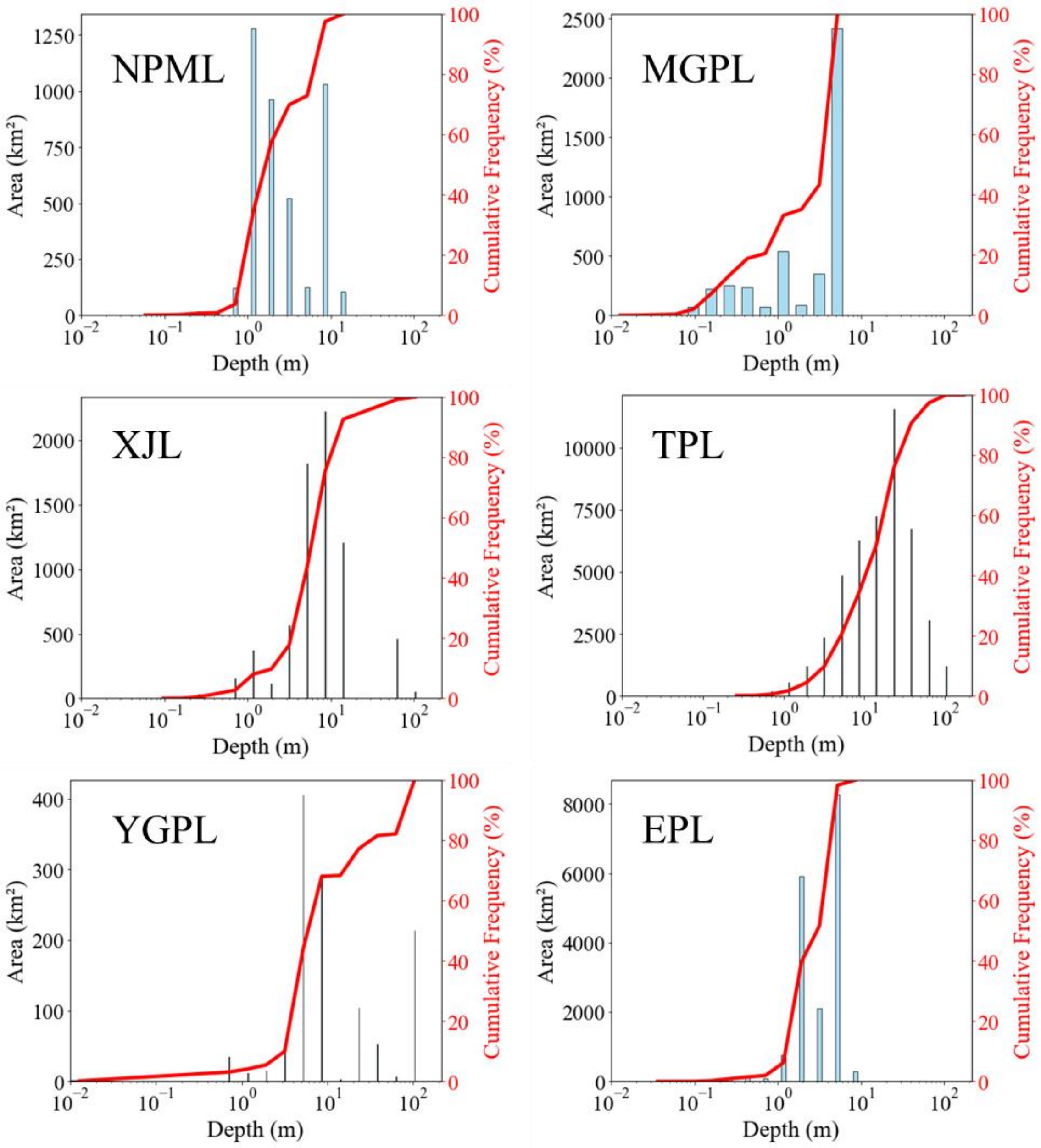

Fig. S5. Cumulative area frequency of lakes at different depth intervals across various lake zones. The histograms represent the total area of lakes within each depth interval.

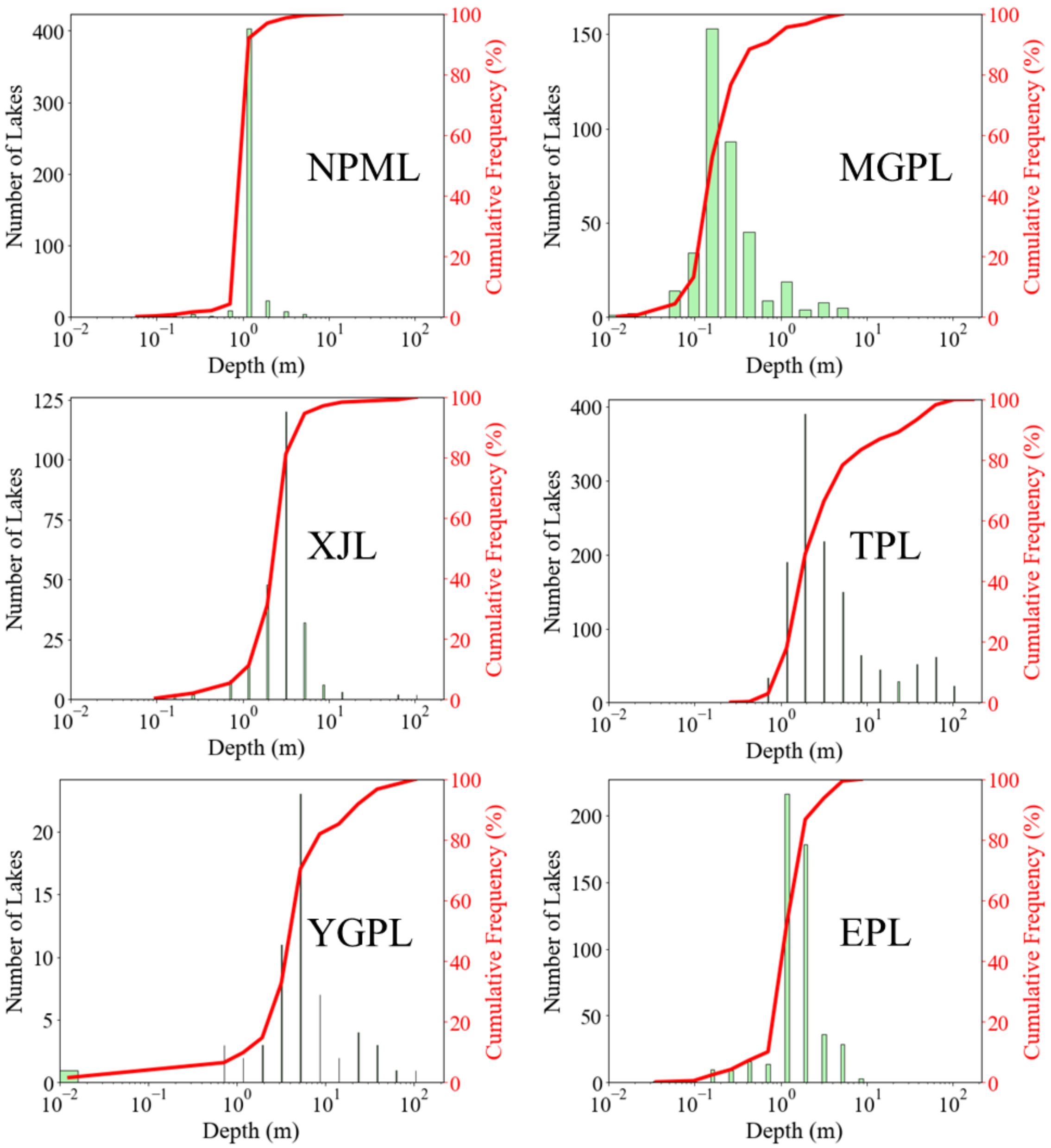

Fig. S6. Cumulative number frequency of lakes at different depth intervals across various lake zones. The histograms represent the total number of lakes within each depth interval.

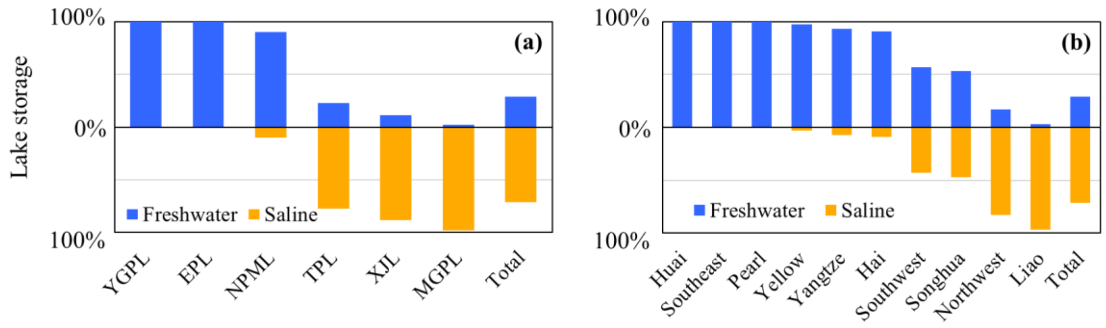

Fig. S7. Proportions of freshwater and saline lake storage by region. The charts are categorized by (a) lake zones and (b) Level-1 watersheds.

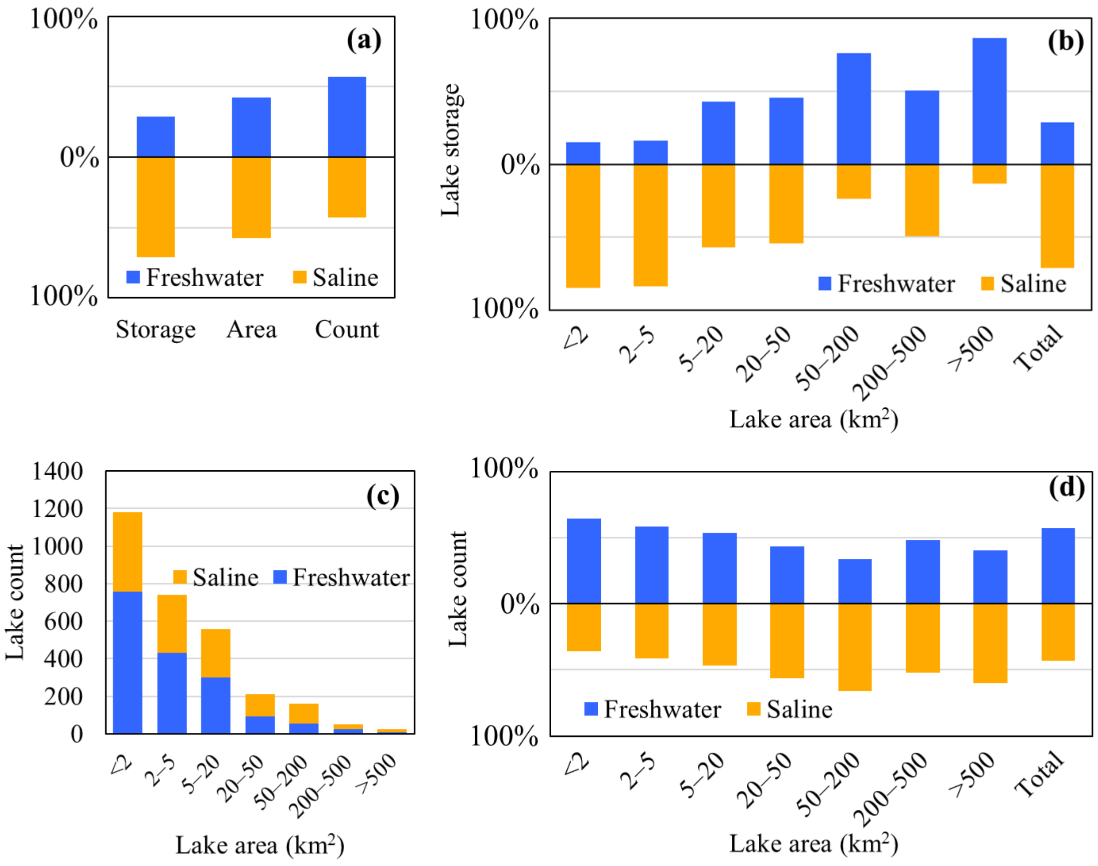

Fig. S8. Proportions of freshwater and saline lake storage by morphologic level. The charts are categorized by (a) Overall proportion by lake storage, lake area, and lake count; (b) Lake storage proportion by lake size levels; (c) Lake count by lake size levels; and (d) Lake count proportion by lake size levels.

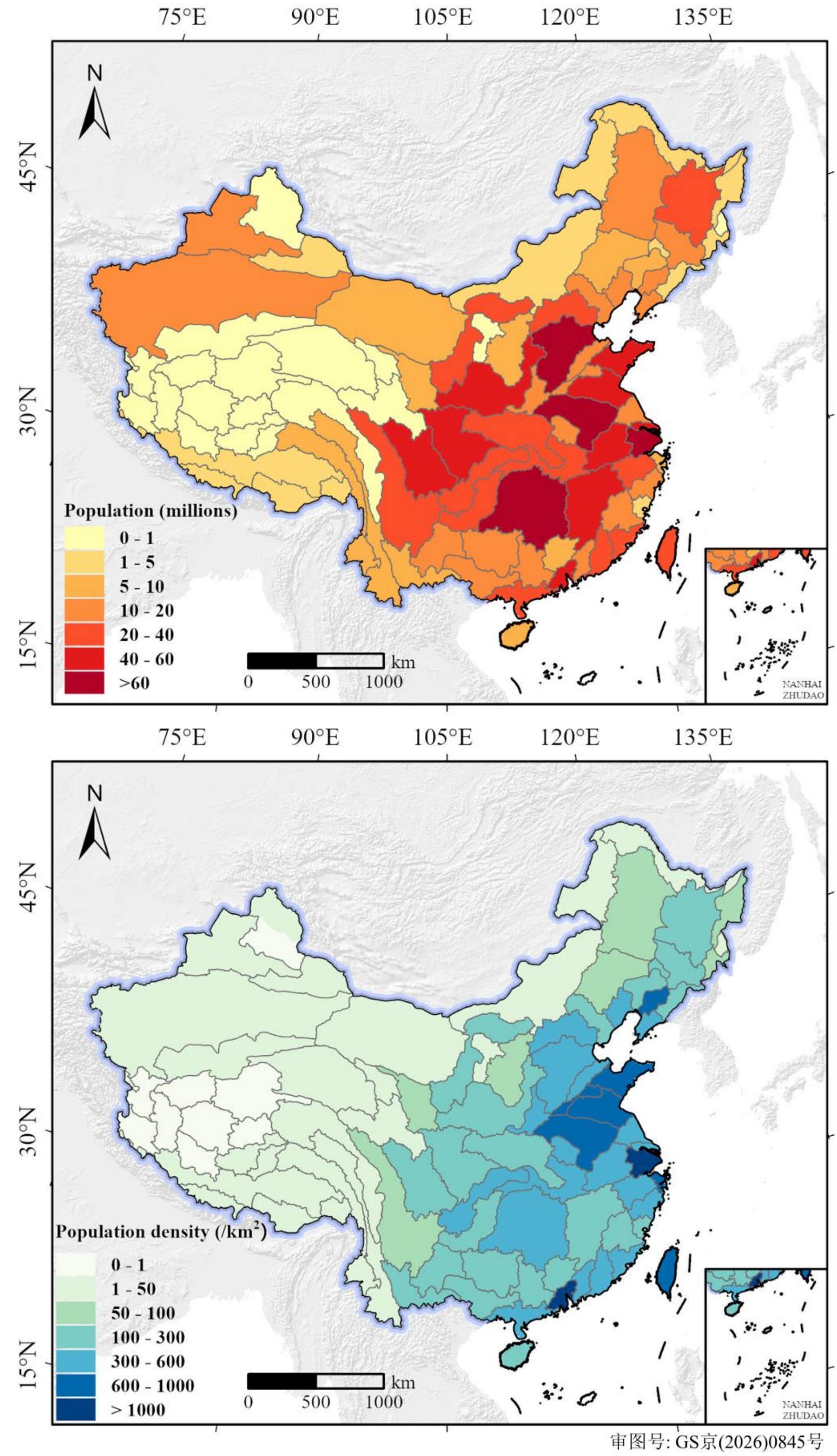

审图号: GS京(2026)0845号

Fig, S9. **Population distribution and population density in 2020 by level-2 watershed across China.**

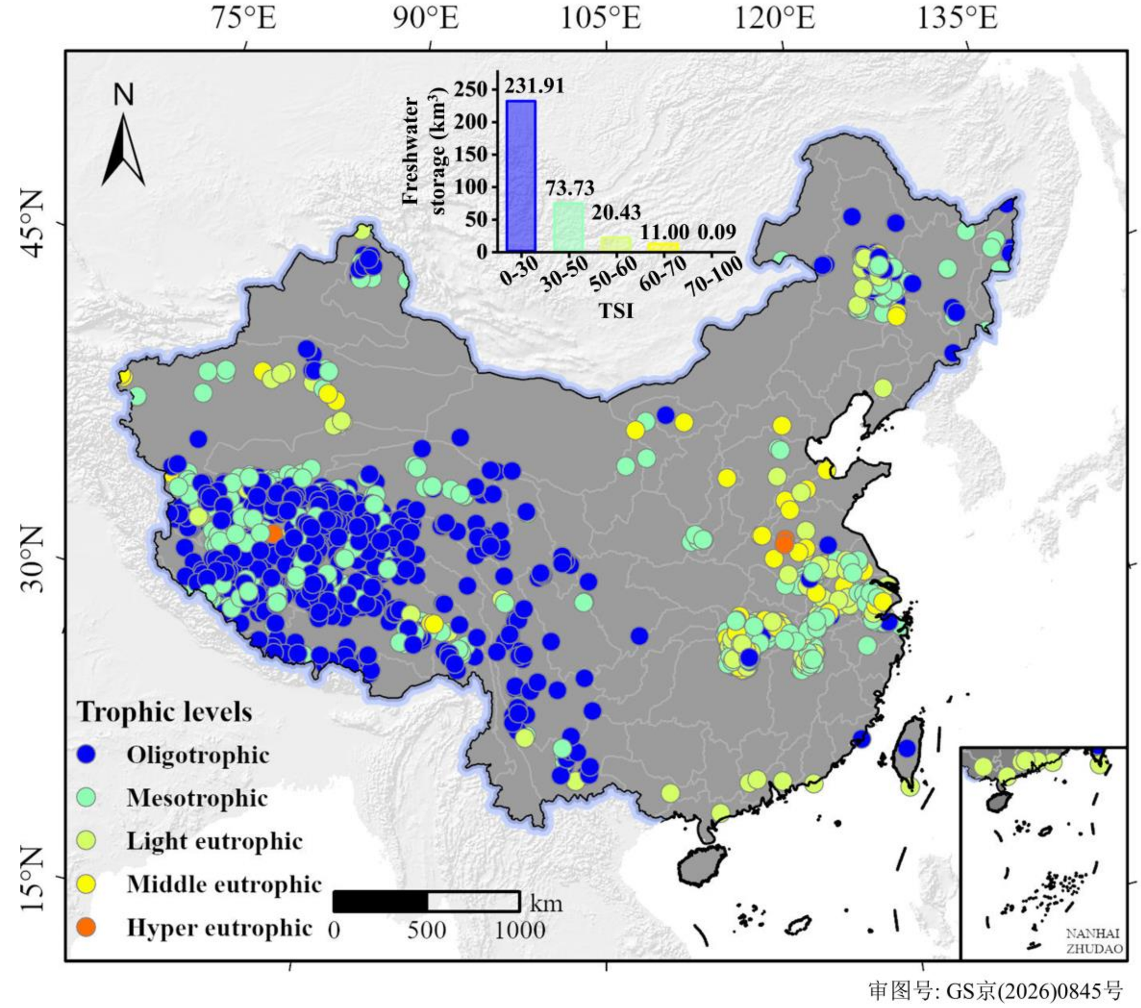

Fig. S10. **Trophic levels of Chinese lakes in circa-2020.** Within the Trophic State Index (TSI) range of 0–100, five trophic state levels are defined: oligotrophic (TSI = 0–30), mesotrophic (TSI = 30–50), light eutrophic (TSI = 50–60), moderate eutrophic (TSI = 60–70), and hyper eutrophic (TSI = 70–100).

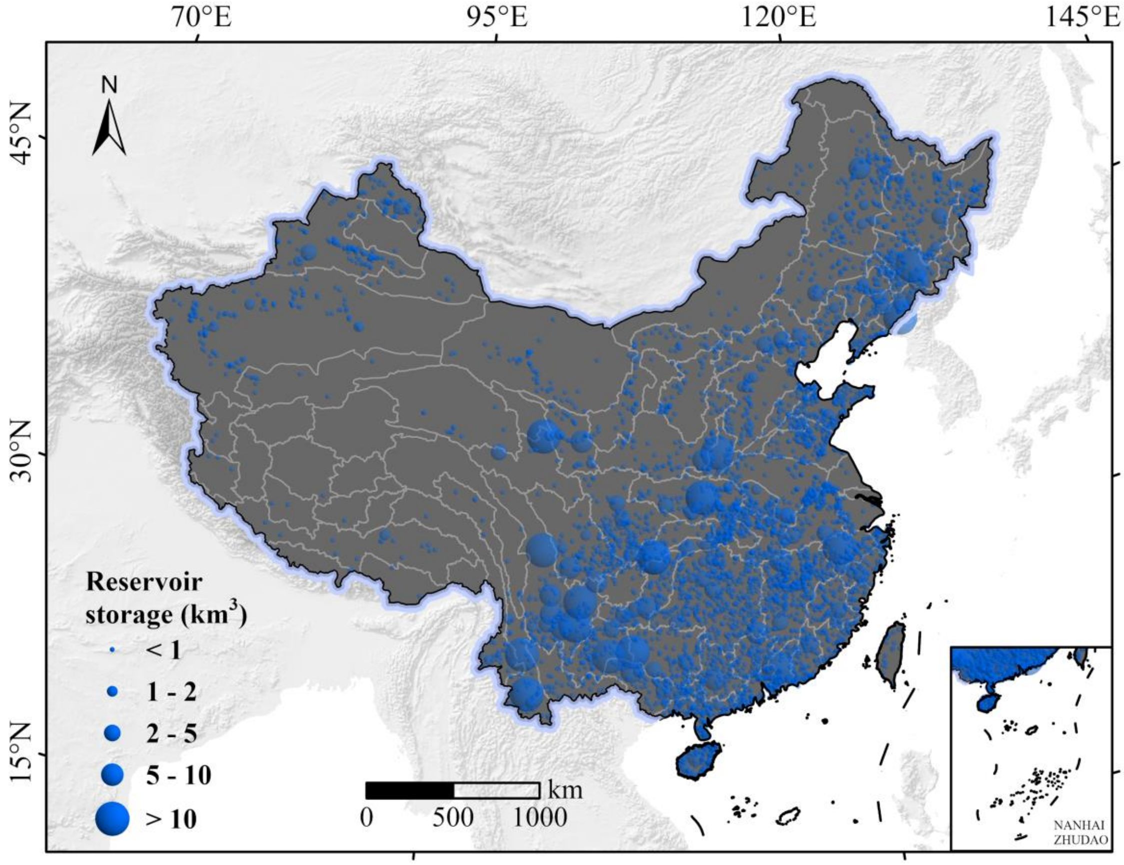

审图号: GS京(2026)0845号

Fig. S11. **Spatial distribution of water storage capacity (volume) of reservoirs larger than 1 km^2 across China.**

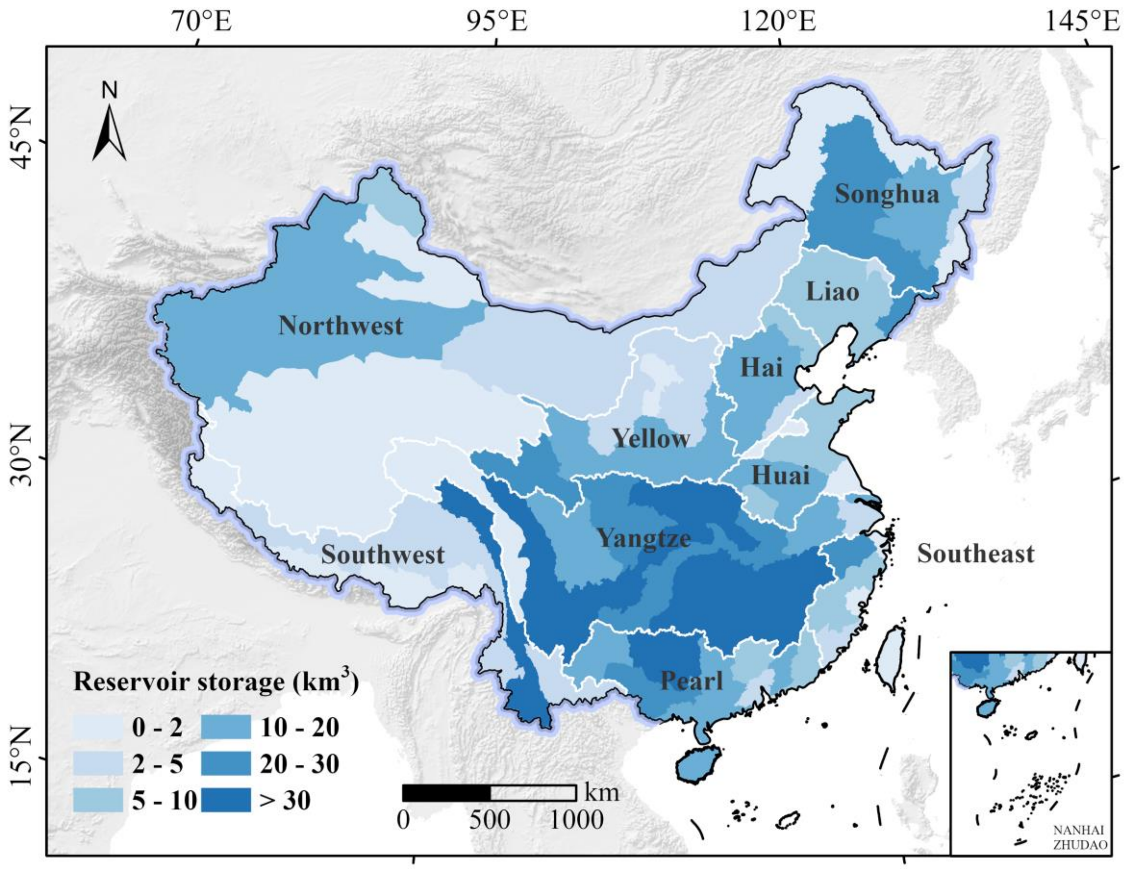

审图号: GS京(2026)0845号

Fig. S12. **Spatial distribution of reservoir water storage capacity (volume) by level-2 watershed** (YZR: Yangtze River, NWR: Northwest Rivers, PR: Pearl River, SWR: Southwest Rivers, YR: Yellow River, SHJR: Songhuajiang River, SER: Southeast Rivers, LR: Liaohe River, HuR: Huaihe River, and HR: Haihe River).

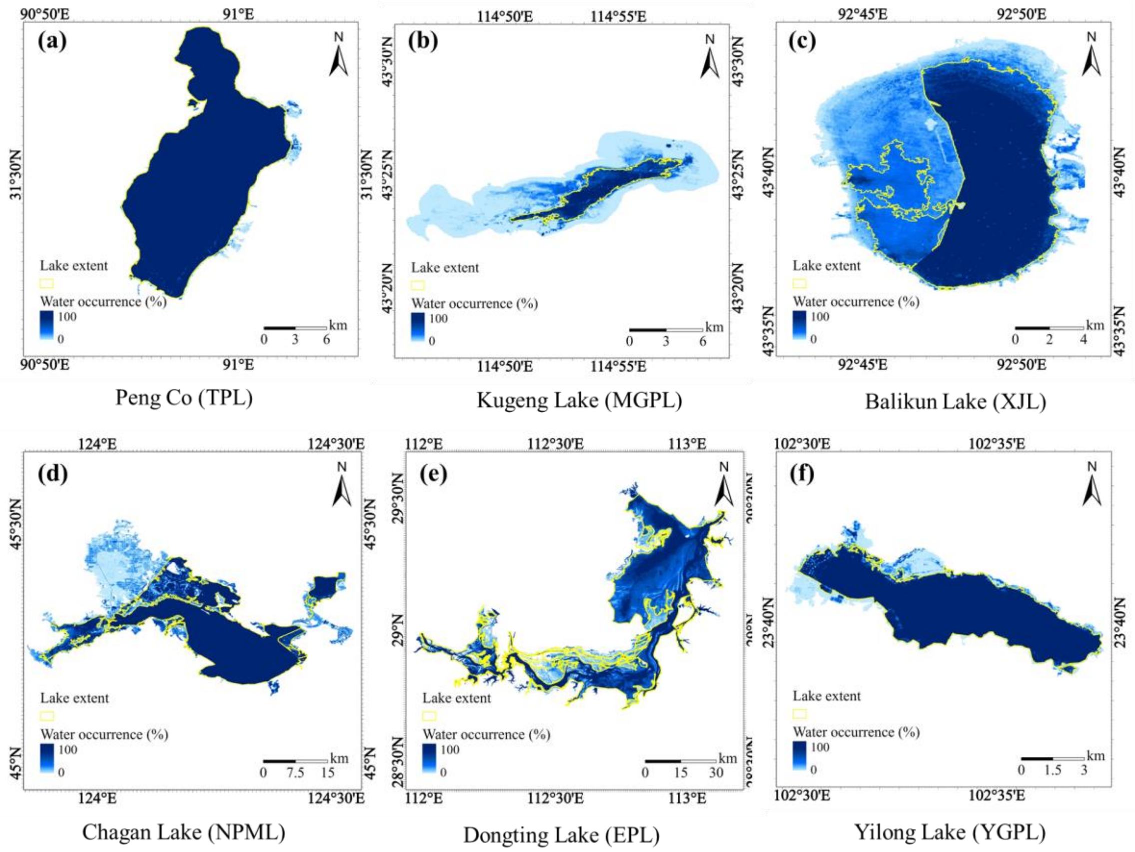

Fig. S13. Illustration of lake water extent extraction using the 25% water occurrence threshold for lakes in different zones (a-f). The raster data illustrate water frequency results derived from the maximum water extent during 1984–2020 and composited over the period 2019–2021. For some shrinking lakes, extensive drawdown zones can be observed around the lake periphery (b). Overall applying a 25% water threshold effectively eliminates the influence of short-lived, low-frequency water occurrences and yields a more stable and representative boundary for delineating lake water extent.

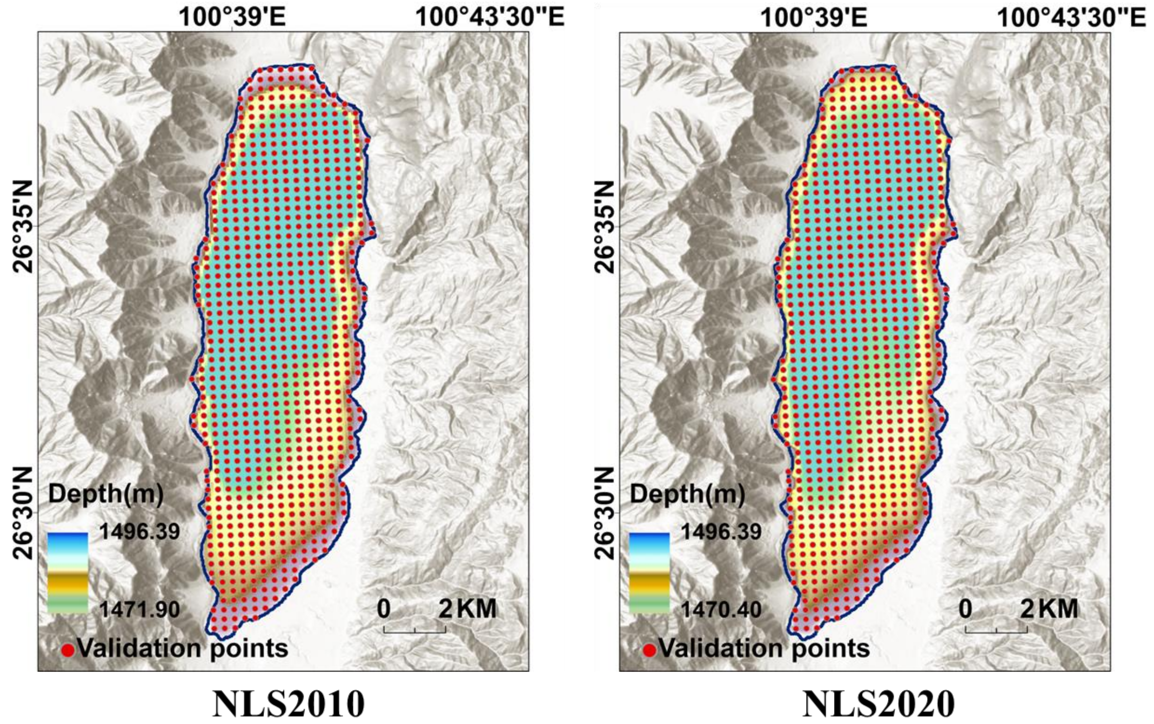

Fig. S14. Elevation accuracy validation of bathymetric data from two survey campaigns, illustrated using Chenghai Lake as an example.

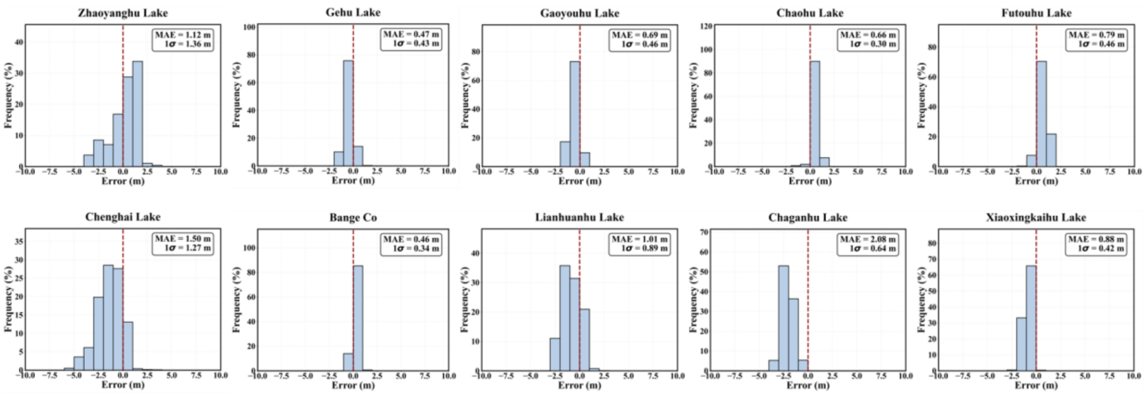

Fig. S15. Bathymetric comparison for ten selected lakes between 2010 and 2020 surveys

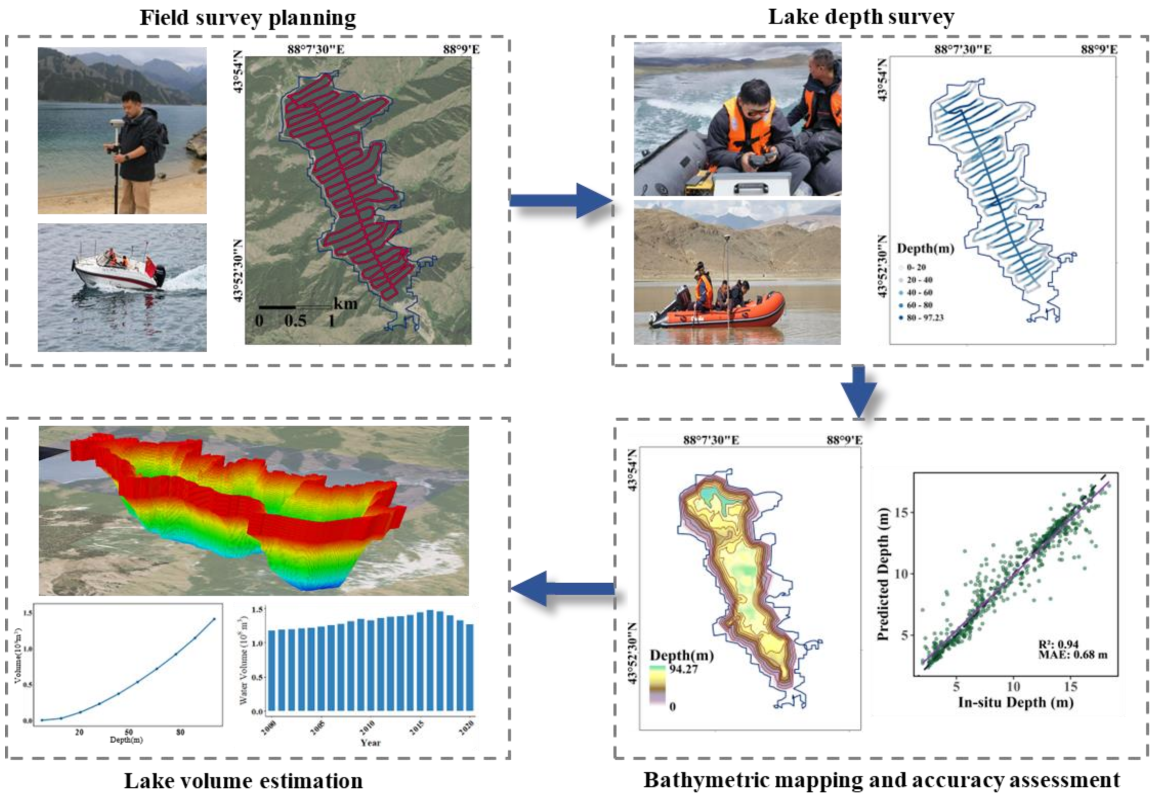

Fig. S16. Technical workflow on field surveying of lake bathymetric data. The flow includes the following steps: (1) Field survey planning: Survey lines are designed based on a field investigation of the target lake to guide water depth measurements; (2) Lake depth survey: An inflatable boat or unmanned surface vehicle (USV) equipped with an echo sounder is used to collect depth data along the planned transects, and a subset of transects is reserved for accuracy validation; (3) Bathymetric mapping and accuracy assessment: A lake bathymetry map is generated using the collected depth data, and the modeling accuracy is evaluated using the validation transects; and (4) Lake storage estimation: A storage–elevation curve is derived from the constructed bathymetry and combined with a time series of remotely sensed water extents to estimate lake water storage and its temporal variation.

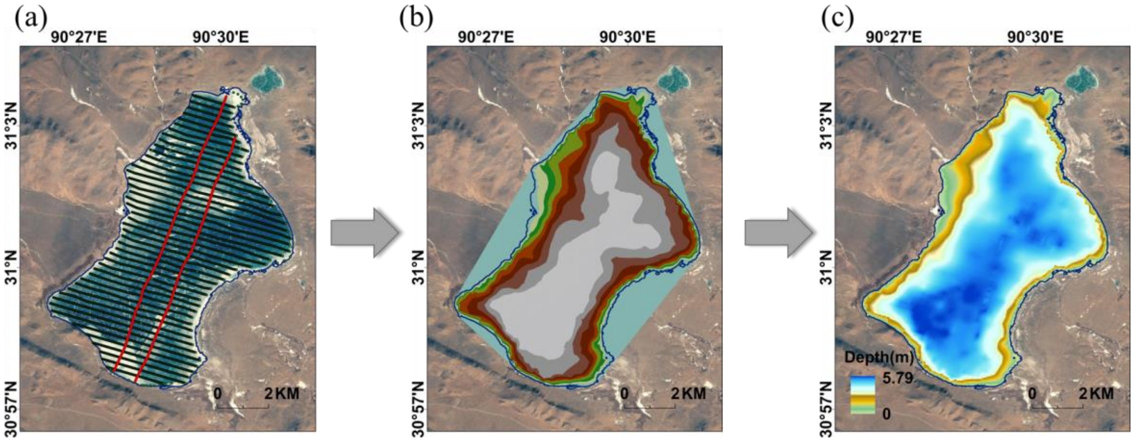

Fig. S17. Technical workflow for generating a raster DEM from bathymetric soundings via TIN construction.

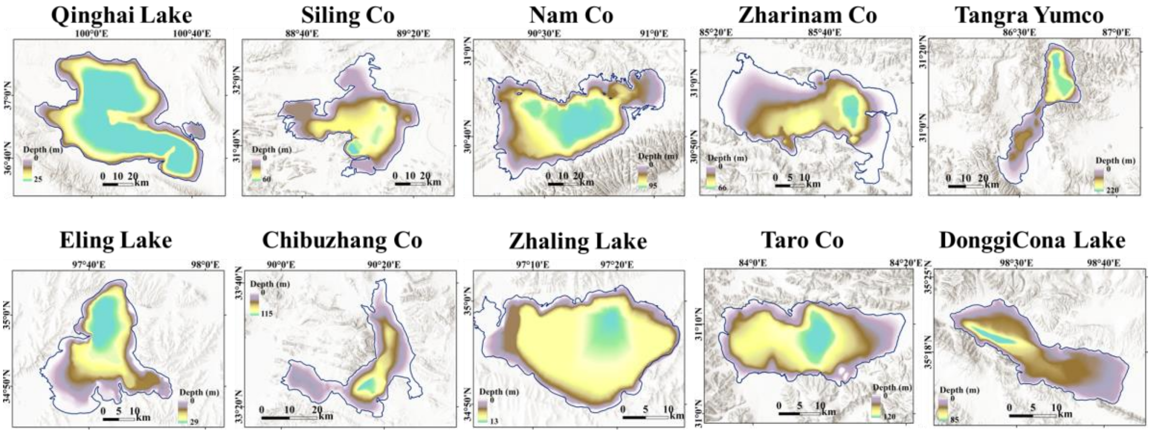

Fig. S18. Bathymetric maps of selected large lakes with field-surveying measurements in the TPL lake zone.

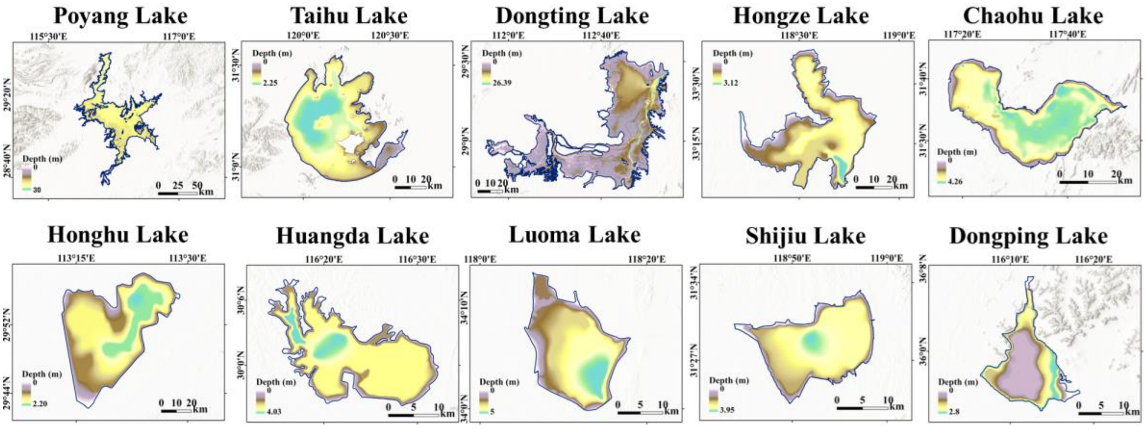

Fig. S19. Bathymetric maps of selected large lakes with field-surveying measurements in the EPL lake zone.

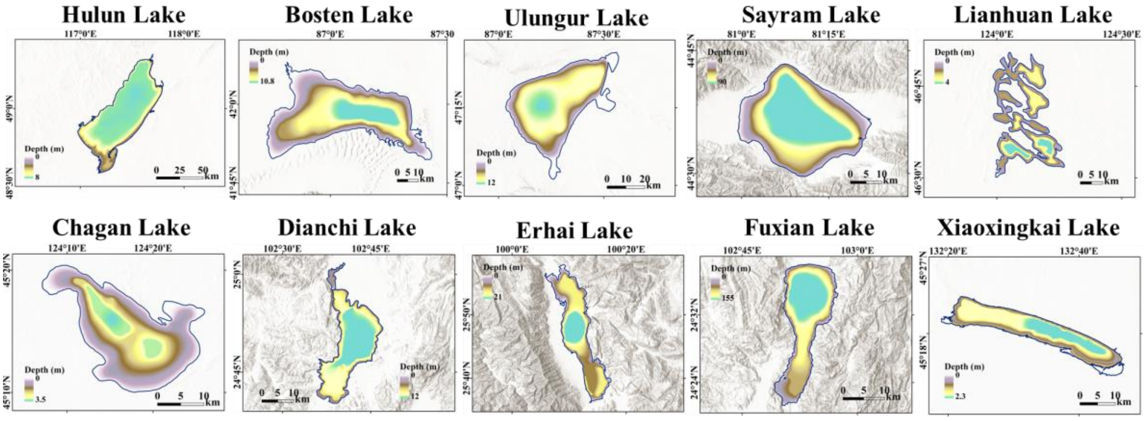

Fig. S20. Bathymetric maps of selected large lakes with field-surveying measurements in the NPML, XJL, MGPL and YGPL lake zones.

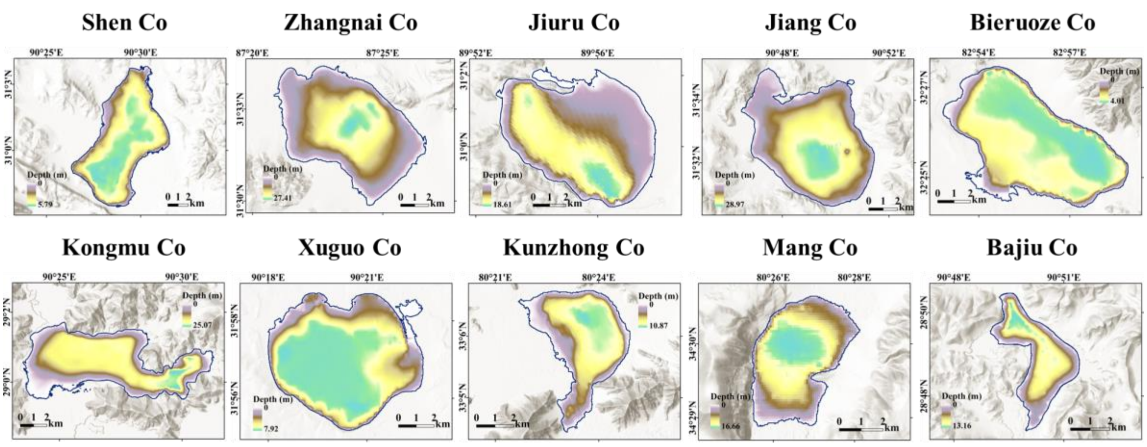

Fig. S21. Bathymetric maps of selected small and medium-sized lakes (< 50 km^2) with field-surveying measurements in the TPL lake zone.

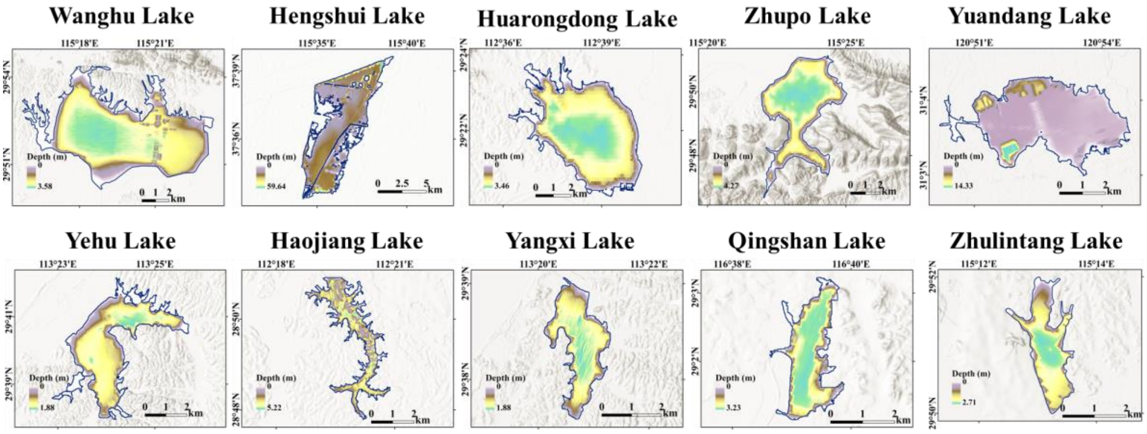

Fig. S22. Bathymetric maps of selected small and medium-sized lakes (< 50 km^2) with field-surveying measurements in the EPL lake zone.

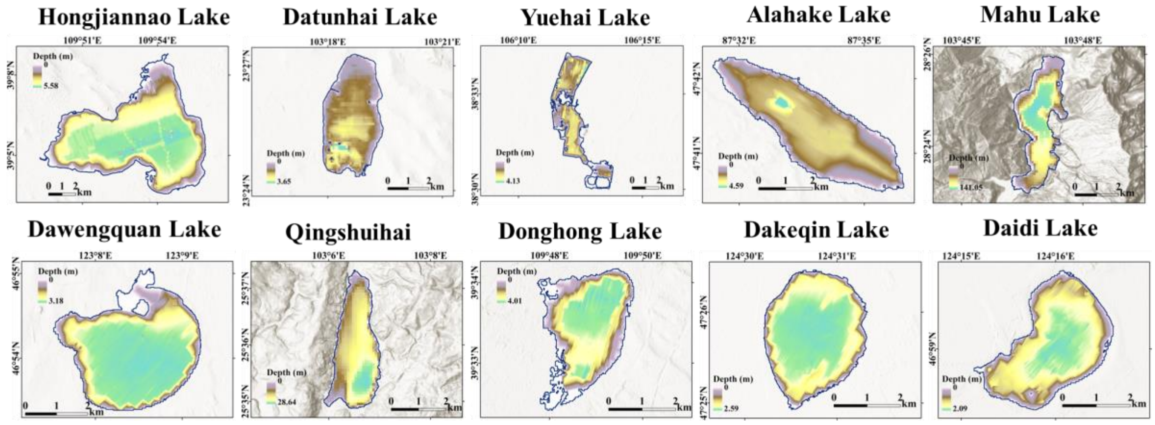

Fig. S23. Bathymetric maps of selected small and medium-sized lakes (< 50 km^2) with field-surveying measurements in the NPML, XJL, MGPL and YGPL lake zones.

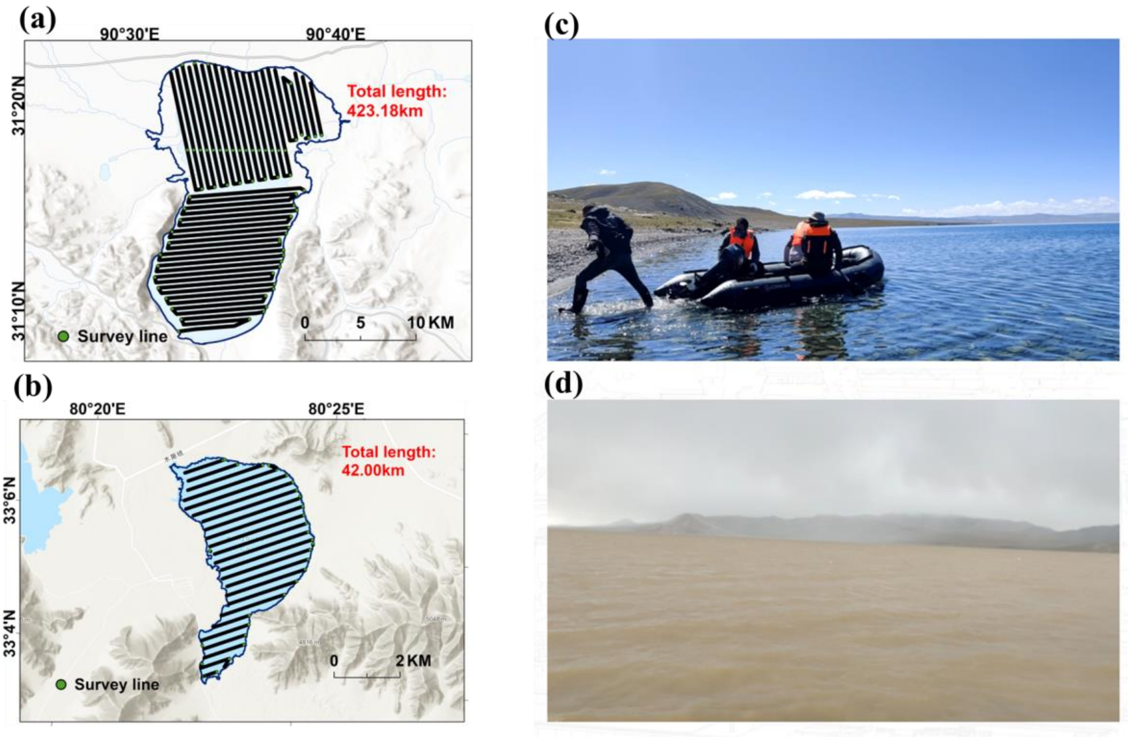

Fig. S24. Schematic illustration of the challenges in bathymetric surveys of lakes on the Tibetan Plateau. (a) Survey lines of Bamucuo (Area > 246.5 km^2; total length: 423.2 km); (b) Survey lines of Kunzhongcuo (Area > 15.5 km^2; total length: 42.0 km); (c–d) Field photographs demonstrating the challenges posed by abrupt weather changes during high-altitude lake surveys on the Tibetan Plateau.

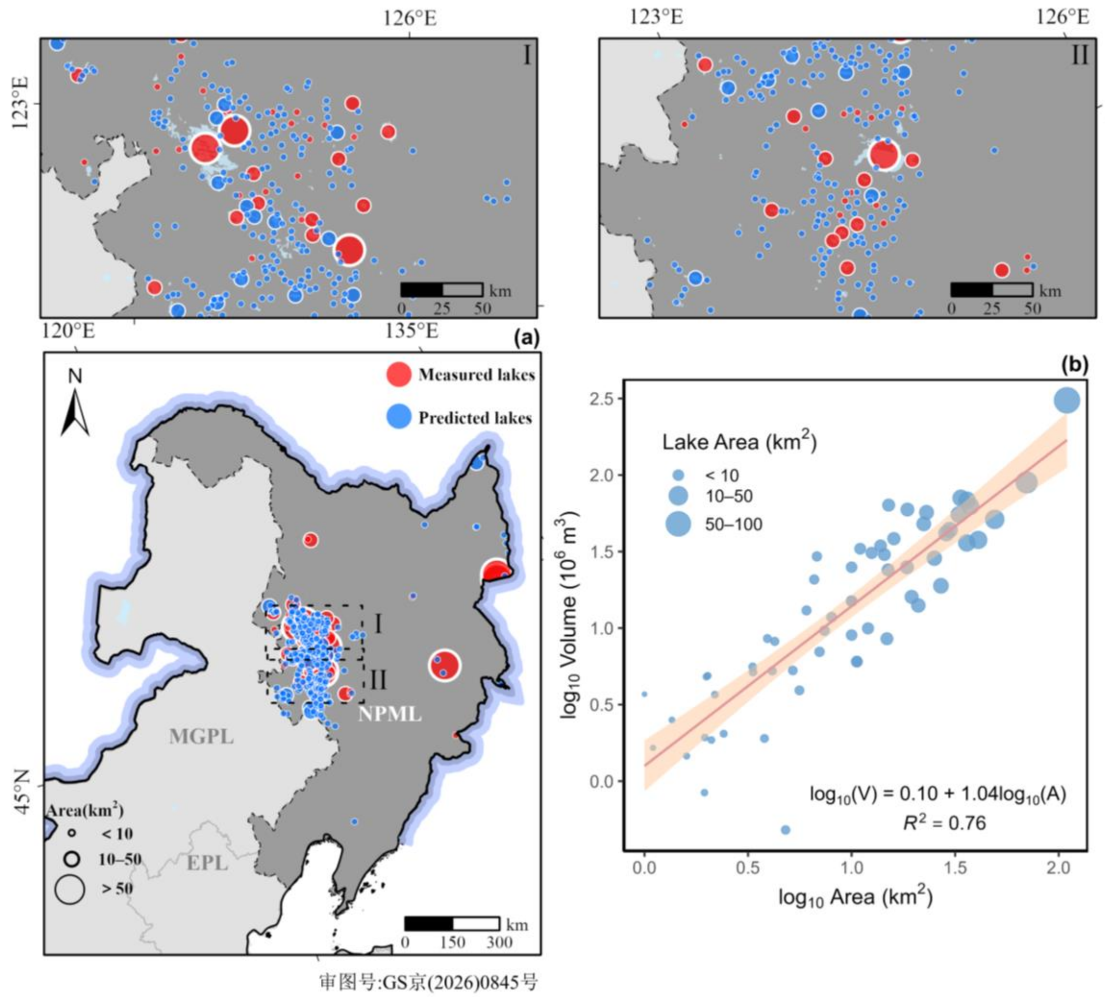

Fig. S25. Distribution of lakes with field-surveyed water storage records and the predictive model used for lake water storage estimation in the NPML lake zone. (a) Spatial distribution of observed and predicted samples. (b) Lake water storage estimation model for this zone.

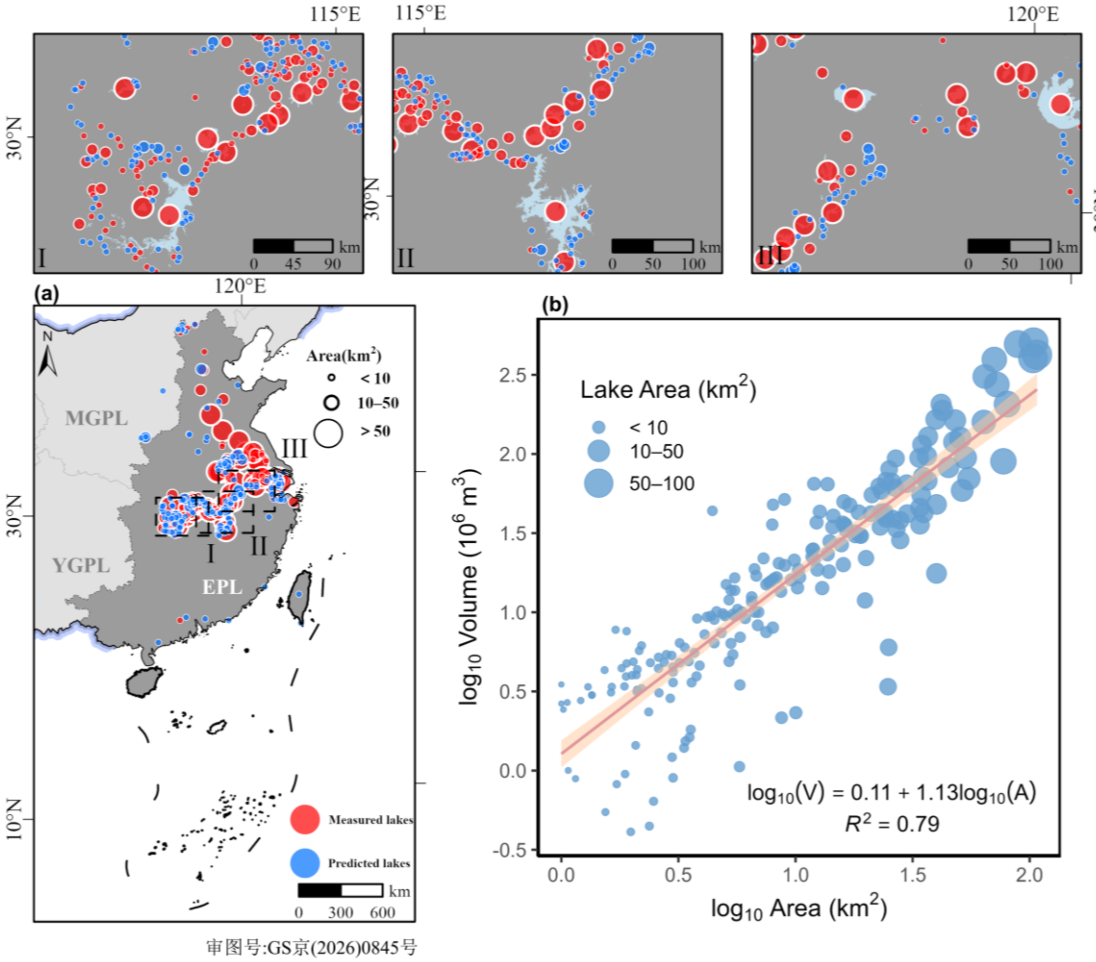

Fig S26. Distribution of lakes with field-surveyed water storage records and the predictive model used for lake water storage estimation in the EPL lake zone. (a) Spatial distribution of observed and predicted samples. (b) Lake water storage estimation model for this zone.

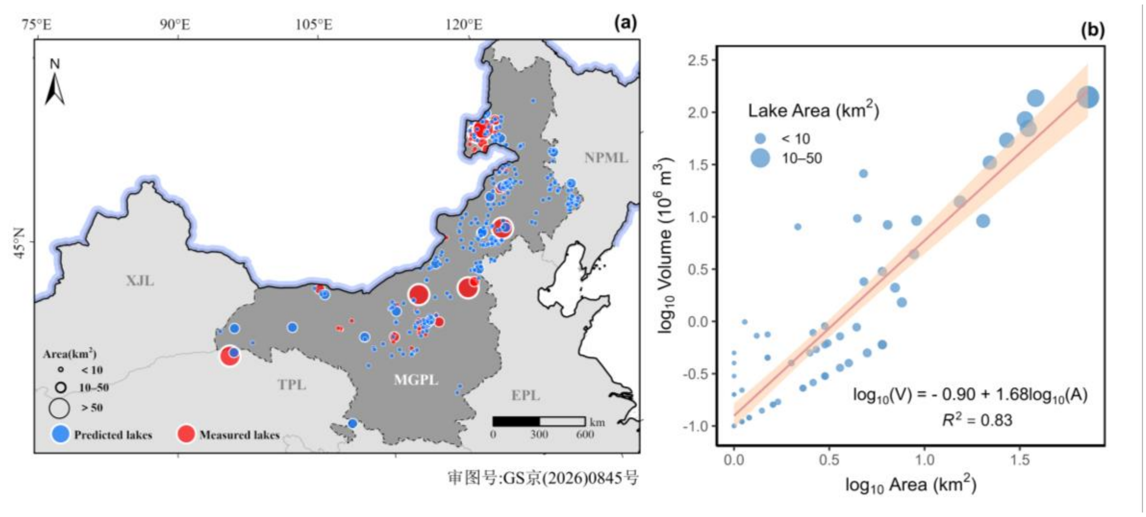

Fig. S27. Distribution of lakes with field-surveyed water storage records and the predictive model used for lake water storage estimation in the MGPL lake zone. (a) Spatial distribution of observed and predicted samples. (b) Lake water storage estimation model for this zone.

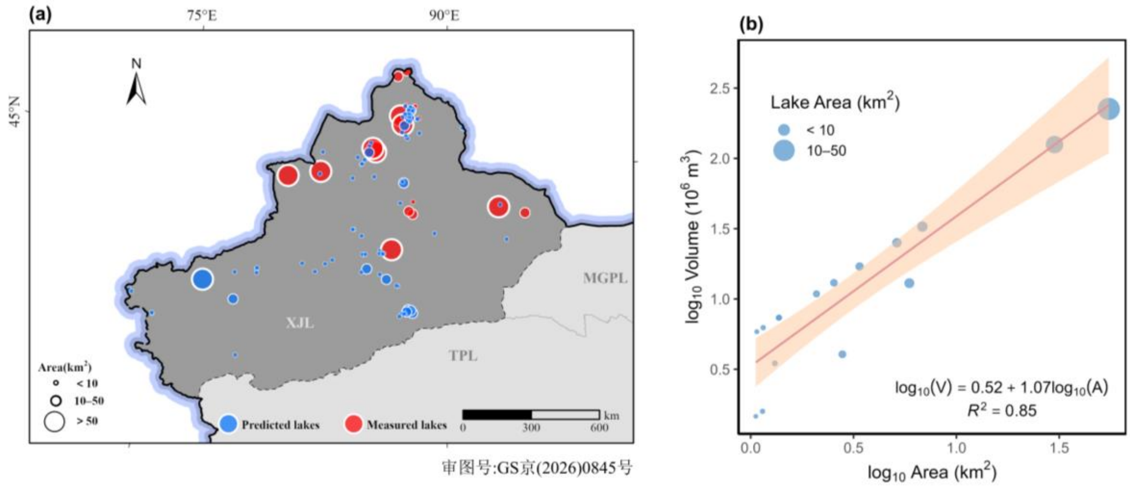

Fig. S28. Distribution of lakes with field-surveyed water storage records and the predictive model used for lake water storage estimation in the XJL lake zone. (a) Spatial distribution of observed and predicted samples. (b) Lake water storage estimation model for this zone.

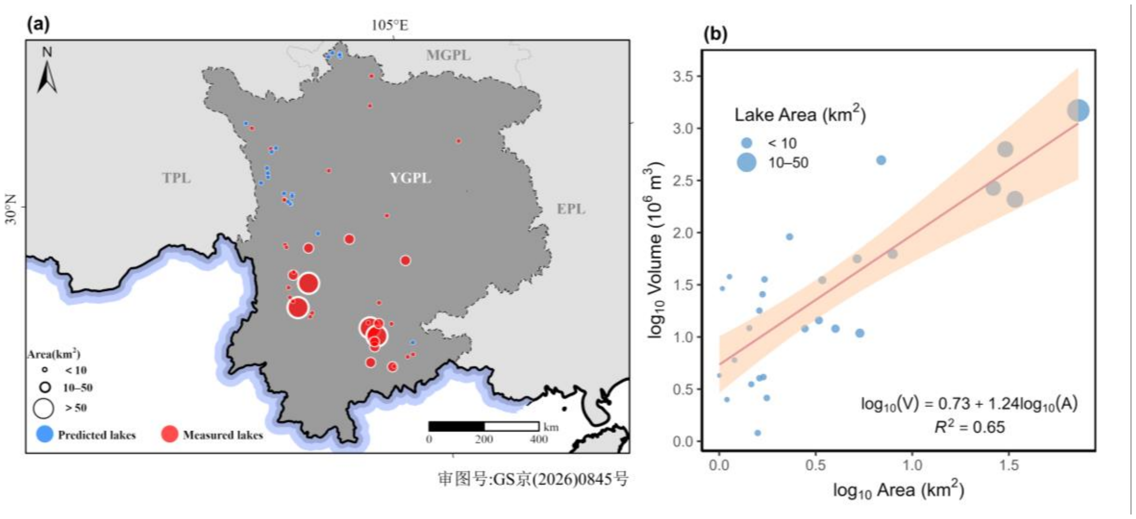

Fig. S29. Distribution of lakes with field-surveyed water storage records and the predictive model used for lake water storage estimation in the YGPL lake zone. (a) Spatial distribution of observed and predicted samples. (b) Lake water storage estimation model for this zone.

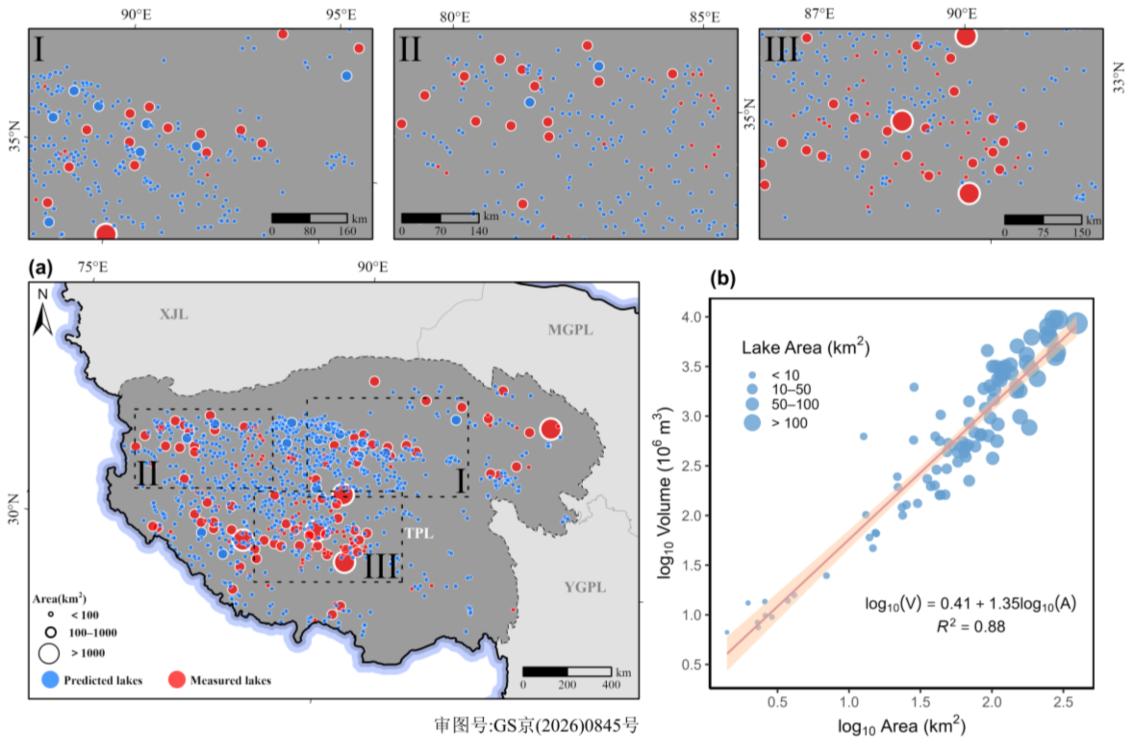

Fig. S30. Distribution of lakes with field-surveyed water storage records and the predictive model used for lake water storage estimation in the TPL lake zone. (a) Spatial distribution of observed and predicted samples. (b) Lake water storage estimation model for this zone.

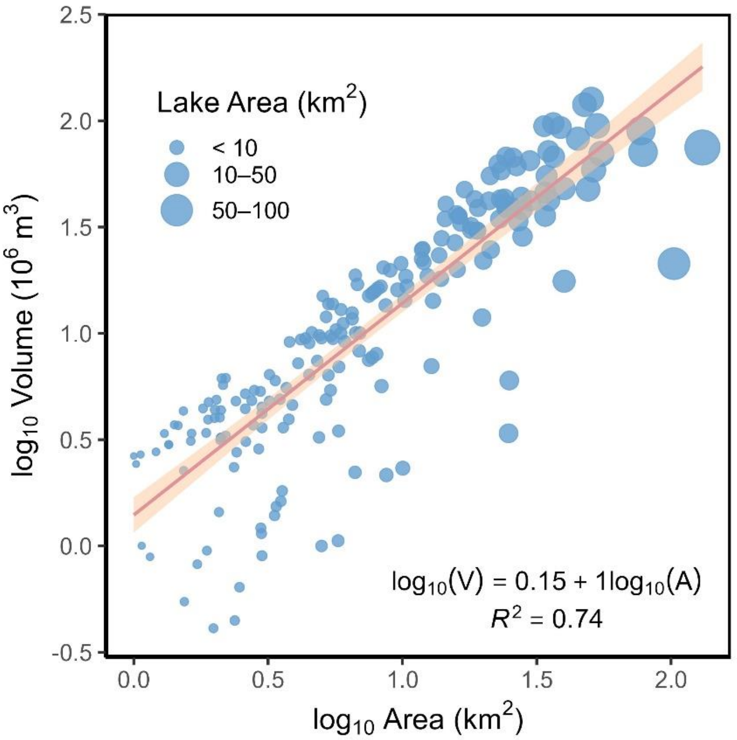

Fig. S31. Log–log area–volume scaling relationship for shallow lakes (< 2 m) in the Eastern Plain Lake region, with increased relative uncertainty (MAPE = 42.8%).

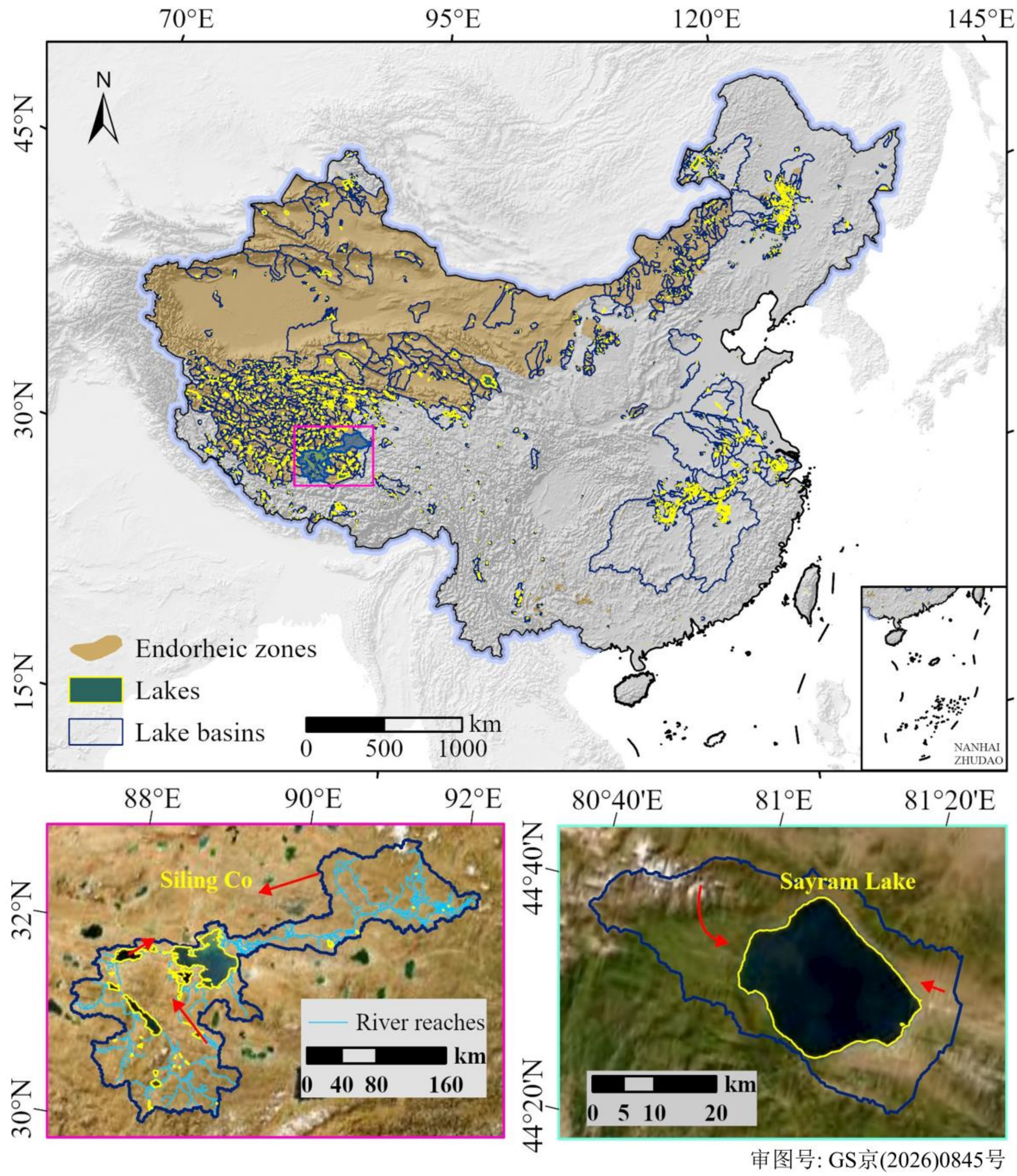

Fig. S32. Map of lake basins and endorheic zones. Subfigures in the bottom panel show typical drainage basin examples where two terminal lakes in these endorheic basins were identified as saline lakes.

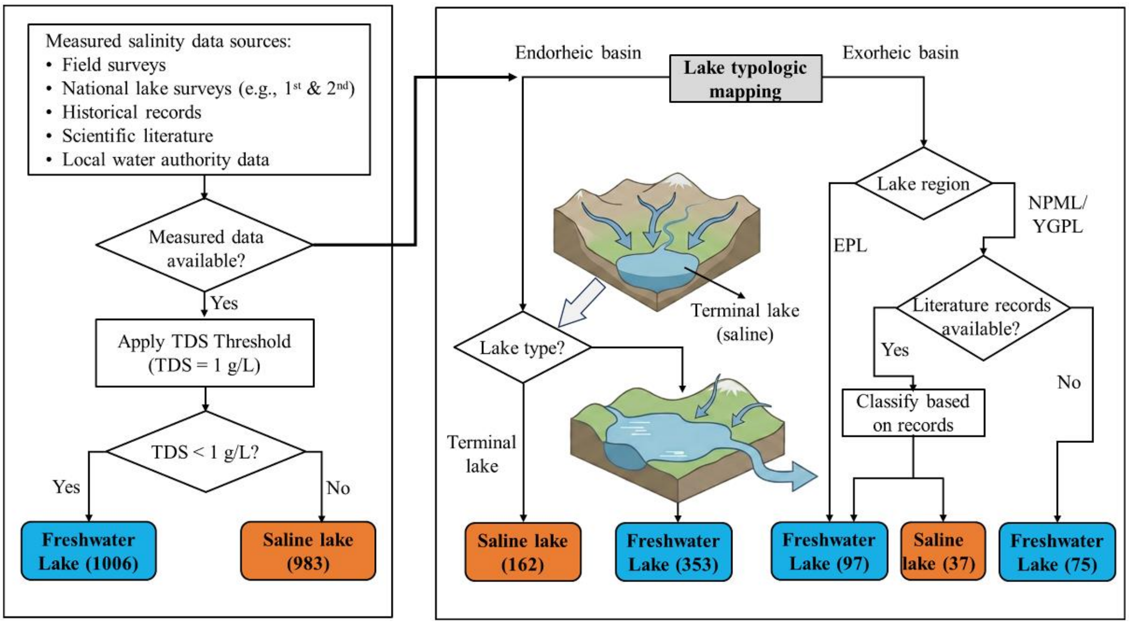

Figure S33. A two-tiered classification framework for distinguishing saline and freshwater lakes in China.

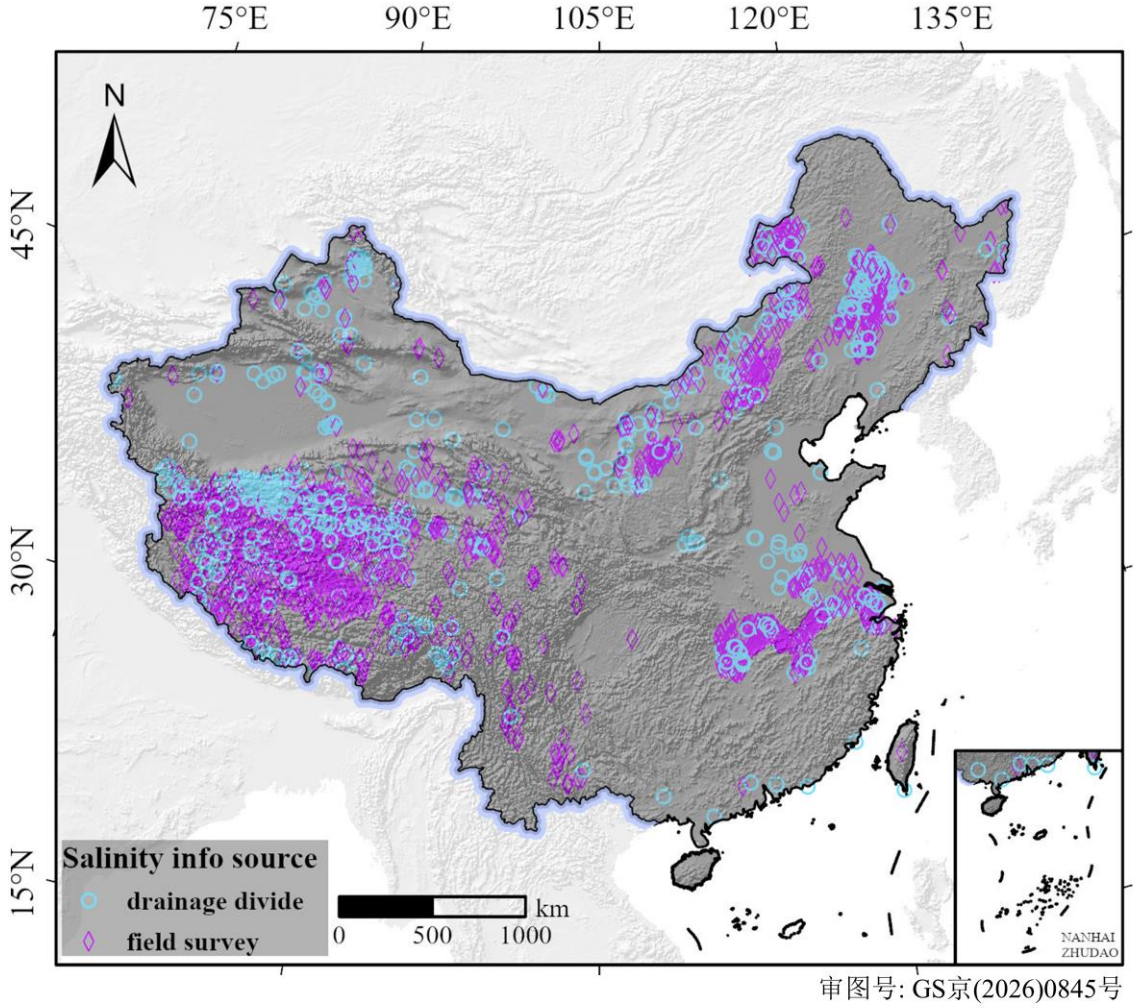

Fig. S34. Distribution map of lakes with saline/freshwater classification based on information from field investigations and drainage division. The type of "drainage divide" refers to lakes where the salinity is primarily judged by topologic relationships within the drainage basin. The type of "field survey" refers to lakes where the typologic classification was double-checked by field-surveyed measurements.

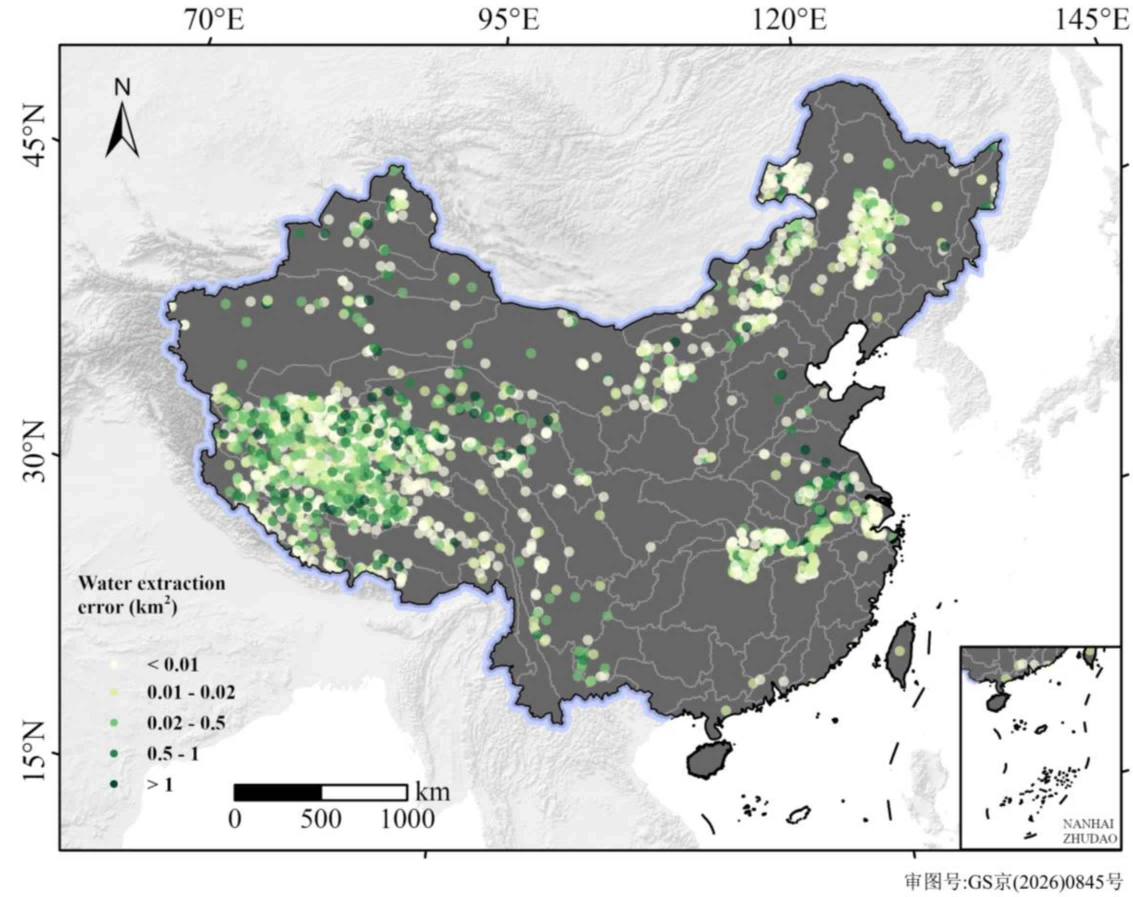

Fig. S35. Spatial distribution of lake mapping uncertainty in the study lakes.

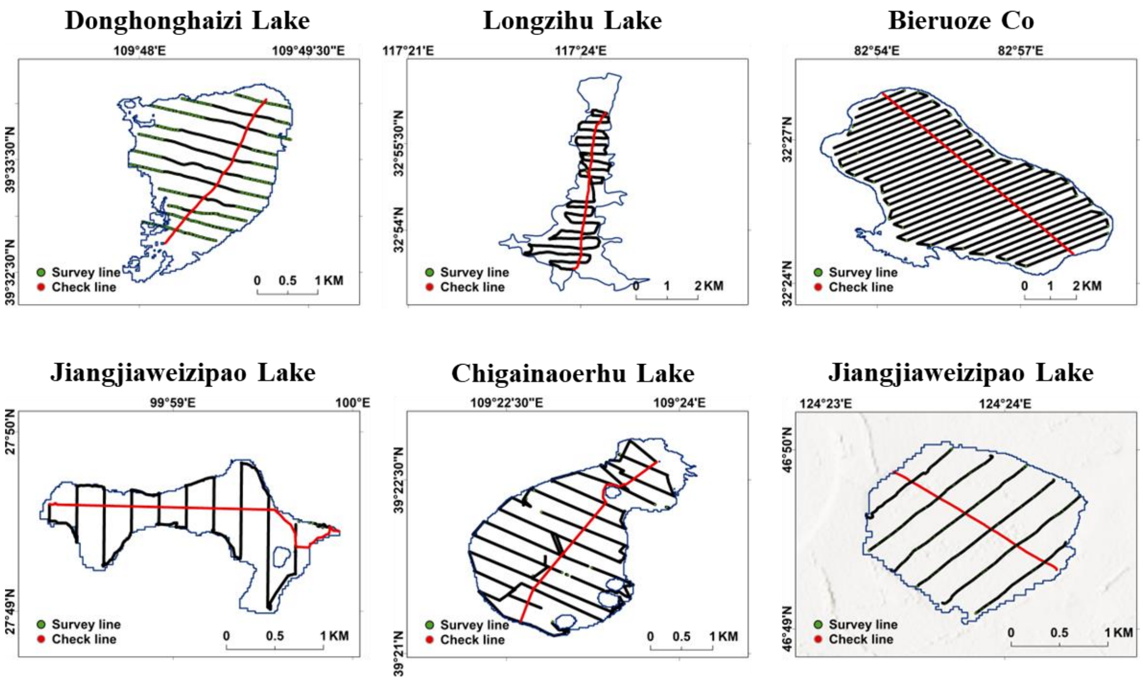

Fig. S36. Schematic diagram of lake bathymetric validation results, where the red lines represent validation (check) lines that were not used in terrain construction.

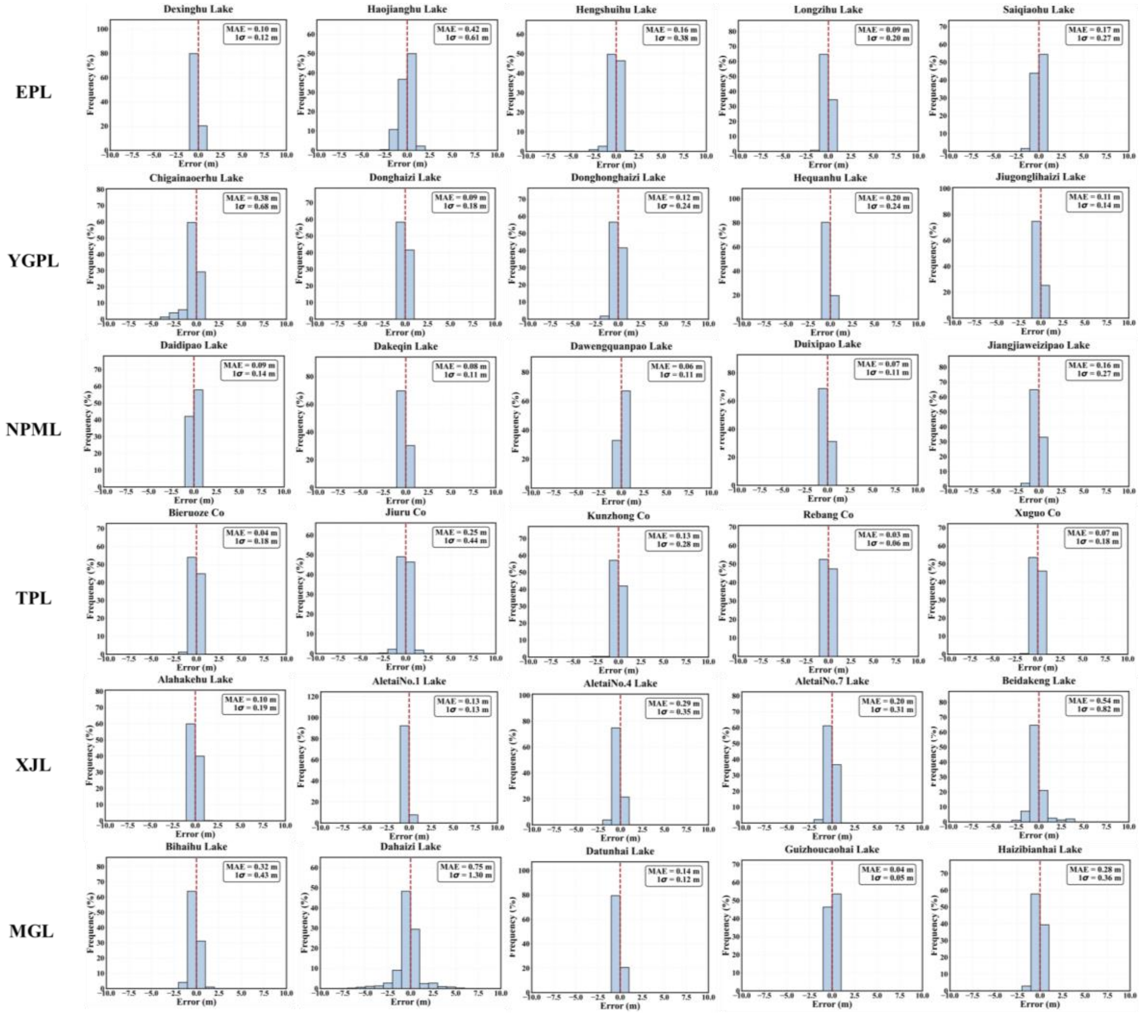

Fig. S37. Five representative lakes were selected in each lake subregion to assess the accuracy of bathymetric measurements.

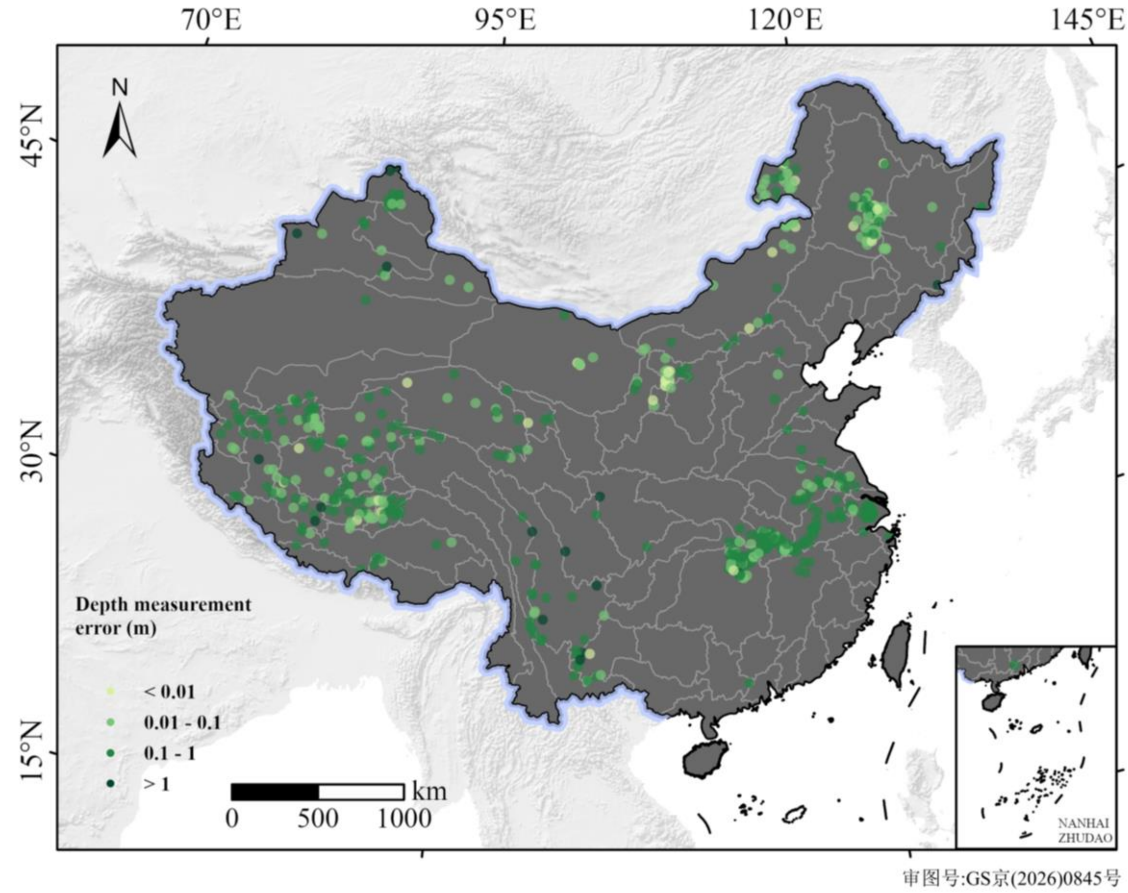

Fig. S38. Spatial distribution of bathymetric depth uncertainty in the study lakes.

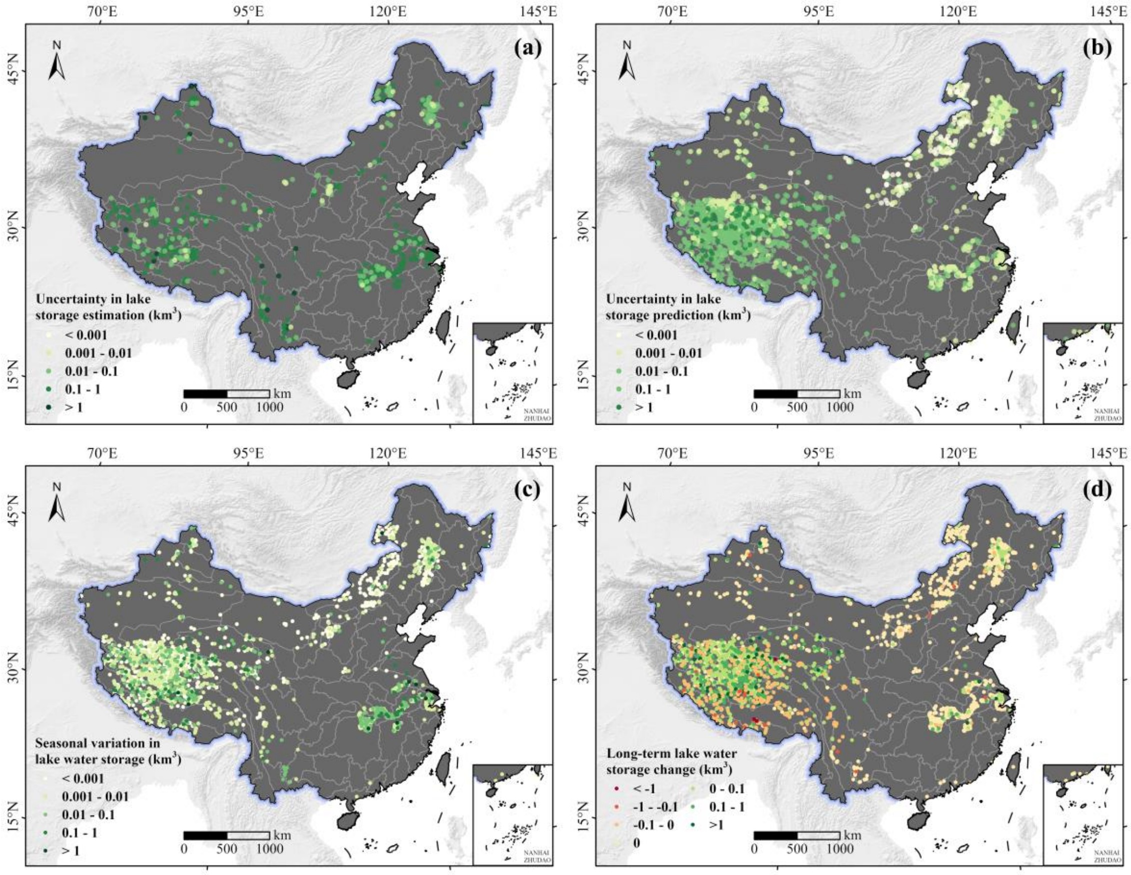

审图号: GS京(2026)0845号

Fig. S39. Uncertainty analysis and temporal variations in lake water storage. (a) The distribution of uncertainty derived from observational data. (b) The distribution of uncertainty introduced by the model. (c) Seasonal variations in lake water storage. (d) Long-term interannual variations in lake water storage over the past two decades.

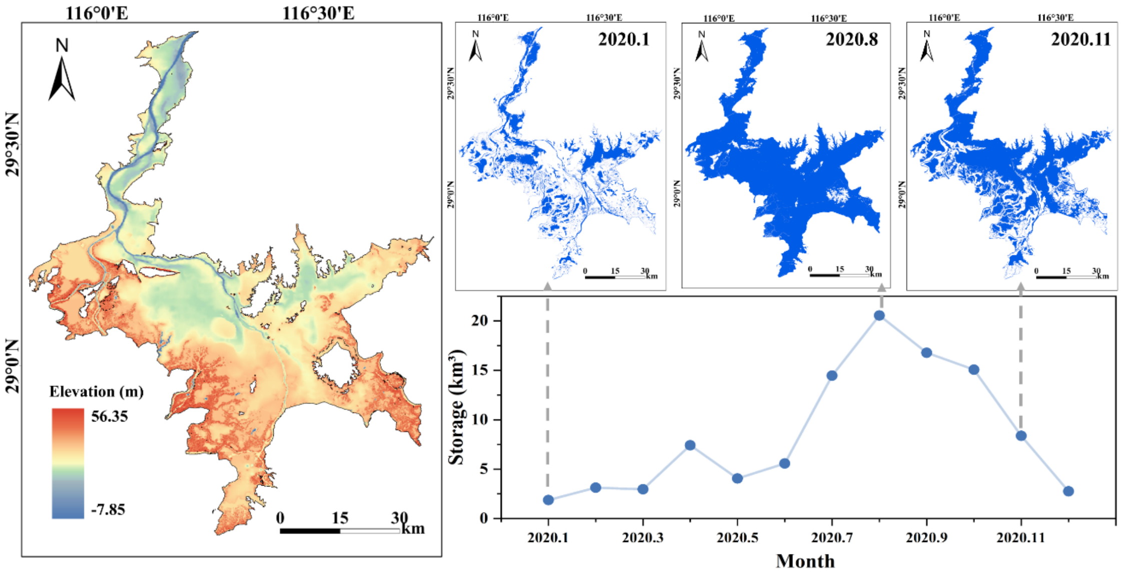

Fig S40. Seasonal variations of Poyang Lake. The left panel displays the lake's bathymetry. The upper right panel visually contrasts the water surface area between January (dry season) and August (wet season), while the line graph below quantifies the seasonal changes in water storage volume throughout the year.

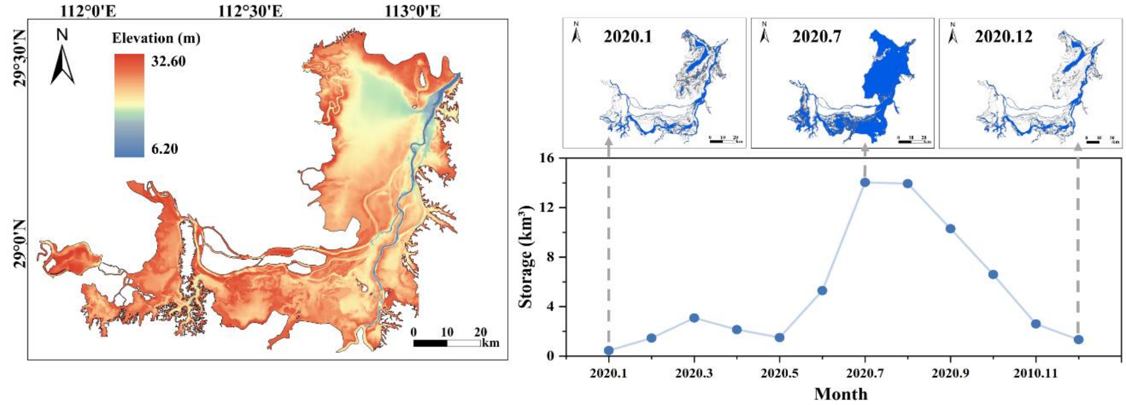

Fig S41. Seasonal variations of Dongting Lake. The left panel presents the lake's bathymetry. The right panels illustrate the seasonal cycle by comparing the water surface extent in January (dry season), July (wet season), and December (dry season), in conjunction with the corresponding monthly water storage curve.

Table S1. Statistics on the count, area, and water storage for lakes with field measurements from different data sources or prediction-based lakes and their corresponding ratios (%) to the national net.

Data source	Count	Count percent	Measured area (km^2)	Area percent	Measured storage (km^3)	Storage percent
NLS-2010	69	2.54%	27,223.73	34.08%	296.74	25.27%
NLS-2020	209	7.70%	26,542.92	33.22%	670.16	57.07%
Literature	310	11.43%	8,749.29	10.95%	58.58	4.99%
Prediction	2,125	78.33%	17,376.46	21.75%	148.83	12.67%
Total	2,713	100.00%	79,892.40	100.00%	1,174.31	100.00%

Note: The row in grey filling color indicates the statistics for the lakes to be predicted.

Table S2. Empirical models for estimating lake water storage in different lake zones.

Lake zone	Statistical estimation function	Sample number	R^2	Bias (%)	MAPE (%)
TPL	log10(V) = 0.41+1.35 log10(A)	120	0.88	-20.60	42.36
YGPL	log10(V) = 0.73+1.24 log10(A)	28	0.65	-26.60	61.06
XJL	log10(V) = 0.52+1.07 log10(A)	16	0.85	+2.20	39.50
MGPL	log10(V)=−0.90+1.68 log10(A)	75	0.83	-21.10	53.91
NPML	log10(V)=0.10+1.04 log10(A)	60	0.76	-15.34	48.73
EPL	log10(V)=0.11+1.13log10(A)	202	0.79	-21.62	35.69
Glacial lakes	log10(V)=1.59+1.45 log10(A)	59	0.91	-4.00	28.26

Note: V represents water storage (10^6 m^3), and A represents surface area (km^2).

Table S3. Statistics on the count, area, and water storage of measured lakes grouped by lake zone and their corresponding ratios (%) to the national net.

Lake zone	Count	Count percent	Area (km^2)	Area percent	Storage (km^3)	Storage percent
NPML	68	16.31%	3,143.65	72.13%	13.77	**89.38%**
EPL	218	45.32%	16,791.10	**94.18%**	63.32	**97.35%**
YGPL	36	64.29%	1,139.93	**96.31%**	29.19	**99.14%**
MGPL	70	20.00%	3,124.33	73.00%	12.73	**94.28%**
XJL	39	17.57%	5,798.82	**82.46%**	80.40	**92.04%**
TPL	157	13.23%	32,518.11	71.93%	826.06	**85.73%**
Total	588	21.67%	62,515.94	**78.25%**	1,025.47	**87.32%**

Note: The items in percentage greater than 75% were marked in bold.

Table S4. Statistics on the count, area, and water storage of measured lakes grouped by size and their corresponding ratios (%) to the national net.

Area range (km^2)	Count	Count percent	Area (km^2)	Area percent	Storage (km^3)	Storage percent
1–50	417	16.86%	4,905.60	30.51%	29.70	29.88%
50–100	44	44.44%	3,193.61	46.10%	28.18	41.30%
>100	127	**90.07%**	54,416.73	**95.66%**	967.59	**96.12%**
Total	588	21.67%	62,515.94	**78.25%**	1,025.47	**87.32%**

Note: The items in percentage greater than 75% were marked in bold.

Table S5. Statistics on the count, area, and water storage of measured lakes grouped by size and their corresponding ratios (%) to the national net for the lake zone NPML.

Area range (km^2)	Count	Count percent	Area (km^2)	Area percent	Storage (km^3)	Storage percent
1–50	59	14.46%	699.36	36.54%	1.93	54.07%
50–100	3	**100.00%**	247.58	**100.00%**	0.58	**100.00%**
>100	6	**100.00%**	2,196.72	**100.00%**	11.26	**100.00%**
Total	68	16.31%	3,143.65	72.13%	13.77	**89.38%**

Note: The items in percentage greater than 75% were marked in bold.

Table S6. Statistics on the count, area, and water storage of measured lakes grouped by size and their corresponding ratios (%) to the national net for the lake zone EPL.

Area range (km^2)	Count	Count percent	Area (km^2)	Area percent	Storage (km^3)	Storage percent
1–50	180	40.63%	1977.43	65.59%	4.46	72.12%
50–100	11	**100.00%**	791.05	**100.00%**	2.58	**100.00%**
>100	27	**100.00%**	14,022.61	**100.00%**	56.28	**100.00%**
Total	218	45.32%	16,791.10	**94.18%**	63.32	**97.35%**

Note: The items in percentage greater than 75% were marked in bold.

Table S7. Statistics on the count, area, and water storage of measured lakes grouped by size and their corresponding ratios (%) to the national net for the lake zone YGPL.

Area range (km^2)	Count	Count percent	Area (km^2)	Area percent	Storage (km^3)	Storage percent
1–50	32	61.54%	328.75	**88.27%**	4.36	**94.49%**
50–100	1	**100.00%**	72.40	**100.00%**	1.48	**100.00%**
>100	3	**100.00%**	738.78	**100.00%**	23.34	**100.00%**
Total	36	64.28%	1,139.93	**96.31%**	29.19	**99.14%**

Note: The items in percentage greater than 75% were marked in bold.

Table S8. Statistics on the count, area, and water storage of measured lakes grouped by size and their corresponding ratios (%) to the national net for the lake zone MGPL.

Area range (km^2)	Count	Count percent	Area (km^2)	Area percent	Storage (km^3)	Storage percent
1–50	64	18.66%	443.35	28.64%	0.73	51.62%
50–100	1	50.00%	51.67	50.39%	0.18	68.30%
>100	5	**100.00%**	2,629.30	**100.00%**	11.81	**100.00%**

Total	70	20.00%	3,124.33	73.00%	12.73	**94.28%**

Note: The items in percentage greater than 75% were marked in bold.

Table S9. Statistics on the count, area, and water storage of measured lakes grouped by size and their corresponding ratios (%) to the national net for the lake zone XJL.

Area range (km^2)	Count	Count percent	Area (km^2)	Area percent	Storage (km^3)	Storage percent
1–50	27	13.04%	258.94	20.31%	7.10	57.48%
50–100	1	25.00%	52.46	19.43%	0.20	10.73%
>100	11	**100.00%**	5,487.42	**100.00%**	73.09	**100.00%**
Total	39	17.57%	5,798.82	**82.46%**	80.40	**92.04%**

Note: The items in percentage greater than 75% were marked in bold.

Table S10. Statistics on the count, area, and water storage of measured lakes grouped by size and their corresponding ratios (%) to the national net for the lake zone TPL.

Area range (km^2)	Count	Count percent	Area (km^2)	Area percent	Storage (km^3)	Storage percent
1–50	55	5.39%	1,197.77	15.06%	11.12	15.60%
50–100	27	34.62%	1,978.46	36.34%	23.14	37.68%
>100	75	**84.27%**	29,341.89	**92.24%**	791.81	**95.30%**
Total	157	13.23%	32,518.11	71.93%	826.06	**85.73%**

Note: The items in percentage greater than 75% were marked in bold.

Table S11. The top twenty highest volume of saltwater lakes across China.

Rank	Lake name	Longitude (°E)	Latitude (°N)	Area (km^2)	Depth (m)	Storage (km^3)	Lake zone	Basin
1	Nam Co	90.6033	30.7383	2019.35	54.90	110.86	TPL	Northwest
2	Qinghai Lake	100.2020	36.8842	4291.30	21.67	92.99	TPL	Northwest
3	Siling Co	88.9932	31.8008	2403.63	30.88	74.22	TPL	Northwest
4	Tangra Yumco	86.6079	31.0687	846.34	85.72	72.55	TPL	Northwest
5	Chibuzhang Co-Duoersuodong Co	90.0538	33.4315	1066.54	33.34	35.55	TPL	Northwest
6	Sayram Lake	81.1699	44.6036	462.39	56.44	26.10	XJL	Northwest
7	Xuru Co	86.4140	30.2942	209.83	121.03	25.40	TPL	Northwest
8	Zhari Namco	85.6125	30.9287	1017.22	24.30	24.72	TPL	Northwest
9	Hala Lake	97.5886	38.2937	618.84	27.32	16.91	TPL	Northwest
10	Yamdrok Yumtso	90.7110	28.9555	546.77	22.95	12.55	TPL	Southwest
11	Ayakkum Lake	89.4609	37.5288	1035.06	10.39	10.75	XJL	Northwest
12	Ulan Ul Lake	90.6054	34.7328	656.42	16.10	10.57	TPL	Northwest
13	Yang Hu	84.5659	35.4054	219.47	46.33	10.17	TPL	Northwest
14	Hulun Lake	117.4000	48.9532	2050.71	4.73	9.70	MGPL	Songhua
15	Peiku Co	85.5835	28.8323	271.08	34.90	9.46	TPL	Southwest
16	Ulungur Lake	87.2893	47.2580	870.86	10.51	9.15	XJL	Northwest
17	Achik-kul Lake	88.4016	37.0773	565.65	15.78	8.93	XJL	Northwest
18	Lumajangdong Co	81.6082	34.0204	392.67	21.95	8.62	TPL	Northwest
19	Dagze Co	87.5180	31.8931	323.75	20.52	6.64	TPL	Northwest
20	Bamu Co	90.5831	31.2644	246.50	26.69	6.58	TPL	Northwest

Table S12. The top twenty highest volume of freshwater lakes across China.

Rank	Lake name	Longitude (°E)	Latitude (°N)	Area (km^2)	Depth (m)	Storage (km^3)	Lake zone	Basin
1	Taro Tso	84.1156	31.1375	488.48	64.71	31.61	TPL	Northwest
2	Fuxian Lake	102.8860	24.5195	213.73	92.51	19.77	YGPL	Pearl
3	Poyang Lake	116.3000	29.0974	3361.70	5.85	19.65	EPL	Yangtze
4	Mapam Yumco	81.4714	30.6848	414.81	43.81	18.17	TPL	Southwest
5	Guozha Co	81.0737	35.0235	248.70	58.10	14.45	TPL	Northwest
6	Wuru Tso	87.9997	31.7163	351.12	40.25	14.13	TPL	Northwest
7	Geren Tso	88.3440	31.1227	475.78	27.62	13.14	TPL	Northwest
8	Eling Lake	97.6992	34.9053	670.43	19.28	12.93	TPL	Yellow
9	Ngangla Ring Tso	83.0777	31.5424	506.14	21.93	11.10	TPL	Northwest
10	Puma Yumco	90.3946	28.5666	291.91	32.05	9.36	TPL	Southwest
11	Donggei Tsona Lake	98.5578	35.2950	247.65	32.11	7.95	TPL	Northwest
12	Dongting Lake	112.8180	29.1057	1718.29	4.18	7.19	EPL	Yangtze
13	Khanka Lake	132.4720	45.2284	1034.09	6.82	7.05	NPML	Songhua
14	Pangong Tso	78.7940	33.7069	492.87	12.53	6.17	TPL	Northwest
15	Taihu Lake	120.1930	31.2011	2340.56	2.39	5.60	EPL	Yangtze
16	Kanas Lake	87.0550	48.8133	44.76	120.04	5.37	XJL	Northwest
17	Gyaring Lake	97.2668	34.9311	552.56	9.32	5.15	TPL	Yellow
18	Beng Co	91.1626	31.2187	143.59	35.65	5.12	TPL	Northwest
19	Chao Lake	117.5350	31.5720	777.87	4.73	3.68	EPL	Yangtze
20	Taiyang Hu	90.6286	35.9262	101.11	31.34	3.17	TPL	Northwest

References

1. Yuan C, Zhan P, Fan C, et al. National estimation of regulated water storage of reservoirs in China. *J Hydrol*. 2024; **645**: 132296.
2. Busker T, de Roo A, Gelati E, et al. A global lake and reservoir volume analysis using a surface water dataset and satellite altimetry. *Hydrol Earth Syst Sci*. 2019; **23**: 669-690.
3. Liu K, Song C, Zhao S, et al. Mapping inundated bathymetry for estimating lake water storage changes from SRTM DEM: A global investigation. *Remote Sens Environ*. 2024; **301**: 113960.
4. Zou Z, Xiao X, Dong J, et al. Divergent trends of open-surface water body area in the contiguous United States from 1984 to 2016. *Proc Natl Acad Sci USA*. 2018; **115**: 3810-3815.
5. Pickens AH, Hansen MC, Hancher M, et al. Mapping and sampling to characterize global inland water dynamics from 1999 to 2018 with full Landsat time-series. *Remote Sens Environ*. 2020; **243**: 111792.
6. Pekel J-F, Cottam A, Gorelick N, Belward AS. High-resolution mapping of global surface water and its long-term changes. *Nature*. 2016; **540**: 418-422.
7. Ma R, Duan H, Hu C, et al. A half - century of changes in China's lakes: Global warming or human influence? *Geophys Res Lett*. 2010; **37**: L24106.
8. Zhu J, Song C, Wang J, Ke L. China’s inland water dynamics: The significance of water body types. *Proc Natl Acad Sci USA*. 2020; **117**: 13876-13878.
9. Liu K, Song C. Modeling lake bathymetry and water storage from DEM data constrained by limited underwater surveys. *J Hydrol*. 2022; **604**: 127260.
10. Messager ML, Lehner B, Grill G, Nedeva I, Schmitt O. Estimating the volume and age of water stored in global lakes using a geo-statistical approach. *Nat Commun*. 2016; **7**: 13603.
11. Heathcote AJ, del Giorgio PA, Prairie YT. Predicting bathymetric features of lakes from the topography of their surrounding landscape. *Can J Fish AquatSci*. 2015; **72**: 643-650.
12. Zhan P, Song C, Liu K, et al. Can we estimate the lake mean depth and volume from the deepest record and auxiliary geospatial parameters? *J Hydrol*. 2023; **617**: 128958.
13. Song C, Fan C, Ma J, Zhan P, Deng X. A spatially constrained remote sensing-based inventory of glacial lakes worldwide. *Sci Data*. 2025; **12**: 464.
14. Song C, Luo S, Liu K, Chen T, Zhang P, Fan C. Widespread declines in water salinity of the endorheic Tibetan Plateau lakes. *Environ Res Commun*. 2022; **4**: 091002.
15. Song K, Shang Y, Wen Z, et al. Characterization of CDOM in saline and freshwater lakes across China using spectroscopic analysis. *Water Res*. 2019; **150**: 403-417.
16. Xu P, Liu K, Shi L, Song C. Machine learning modeling reveals the spatial variations of lake water salinity on the endorheic Tibetan Plateau. *J Hydrol: Reg Stud*. 2024; **56**: 102042.
17. Lehner B, Grill G. Global river hydrography and network routing: baseline data and new approaches to study the world's large river systems. *Hydrol Processes*. 2013; **27**: 2171-2186.
18. Liu K, Song C, Ke L, Jiang L, Ma R. Automatic watershed delineation in the Tibetan endorheic basin: A lake-oriented approach based on digital elevation models. *Geomorphology*. 2020; **358**: 107127.
19. Sikder MS, Wang J, Allen GH, et al. Lake-TopoCat: a global lake drainage topology and catchment database. *Earth Syst Sci Data*. 2023; **2023**: 1-42.
20. Song L, Song C, Luo S, et al. Integrating ICESat-2 altimetry and machine learning to

estimate the seasonal water level and storage variations of national-scale lakes in China. *Remote Sens Environ*. 2023; **294**: 113657.
21. Liu L, Cao X, Li S, Jie N. A 31-year (1990–2020) global gridded population dataset generated by cluster analysis and statistical learning. *Sci Data*. 2024; **11**: 124.
22. Hu M, Ma R, Xue K, et al. A dataset of trophic state index for nation-scale lakes in China from 40-year Landsat observations. *Sci Data*. 2024; **11**: 659.
